# Synergistic “Anchor-Capture” Enabled by Amino and Carboxyl for Constructing Robust Interface of Zn Anode

**DOI:** 10.1007/s40820-023-01171-w

**Published:** 2023-08-28

**Authors:** Zhen Luo, Yufan Xia, Shuang Chen, Xingxing Wu, Ran Zeng, Xuan Zhang, Hongge Pan, Mi Yan, Tingting Shi, Kai Tao, Ben Bin Xu, Yinzhu Jiang

**Affiliations:** 1 School of Materials Science and Engineering, Zhejiang University, Hangzhou, 310027 People’s Republic of China; 2 ZJU-Hangzhou Global Scientific and Technological Innovation Center, Zhejiang University, Hangzhou, 311215 People’s Republic of China; 3 State Key Laboratory of Fluid Power and Mechatronic Systems, Key Laboratory of Advanced Manufacturing Technology of Zhejiang Province, School of Mechanical Engineering, Zhejiang University, Hangzhou, 310027 People’s Republic of China; 4 Institute of Science and Technology for New Energy, Xi’an Technological University, Xi’an, 710021 People’s Republic of China; 5 State Key Laboratory of Baiyunobo Rare Earth Resource Researches and Comprehensive Utilization, Baotou Research Institute of Rare Earths, Baotou, 014030 People’s Republic of China; 6 Guangdong Provincial Engineering Technology Research Center of Vacuum Coating Technologies and New Energy Materials, Department of Physics, Jinan University, Guangzhou, 510632 Guangdong People’s Republic of China; 7 Zhejiang-Israel Joint Laboratory of Self-Assembling Functional Materials, Hangzhou, 311200 People’s Republic of China; 8 Mechanical and Construction Engineering, Faculty of Engineering and Environment, Northumbria University, Newcastle upon Tyne, NE1 8ST UK

**Keywords:** Zn anode–electrolyte interface, Polar groups, Synergistic “anchor-capture” effect, Side reactions, Dendrite growth

## Abstract

While the rechargeable aqueous zinc-ion batteries (AZIBs) have been recognized as one of the most viable batteries for scale-up application, the instability on Zn anode–electrolyte interface bottleneck the further development dramatically. Herein, we utilize the amino acid glycine (Gly) as an electrolyte additive to stabilize the Zn anode–electrolyte interface. The unique interfacial chemistry is facilitated by the synergistic “anchor-capture” effect of polar groups in Gly molecule, manifested by simultaneously coupling the amino to anchor on the surface of Zn anode and the carboxyl to capture Zn^2+^ in the local region. As such, this robust anode–electrolyte interface inhibits the disordered migration of Zn^2+^, and effectively suppresses both side reactions and dendrite growth. The reversibility of Zn anode achieves a significant improvement with an average Coulombic efficiency of 99.22% at 1 mA cm^−2^ and 0.5 mAh cm^−2^ over 500 cycles. Even at a high Zn utilization rate (depth of discharge, DOD_Zn_) of 68%, a steady cycle life up to 200 h is obtained for ultrathin Zn foils (20 μm). The superior rate capability and long-term cycle stability of Zn–MnO_2_ full cells further prove the effectiveness of Gly in stabilizing Zn anode. This work sheds light on additive designing from the specific roles of polar groups for AZIBs.

**Supplementary Information:**

The online version contains supplementary material available at 10.1007/s40820-023-01171-w.

## Highlights

- The synergistic “anchor-capture” mechanism of polar groups on Zn stripping/plating process is firstly proposed.
- The amino group firmly anchors on Zn surface and the carboxyl group strongly captures Zn^2+^, constructing a robust anode–electrolyte interface and inducing uniform Zn deposition.
- The ultra-stable cycle lifespan of Zn–Zn symmetric cell (over 2800 h) and high utilization rate of Zn anode (the depth of discharge up to 68% for 200 h) are achieved under the proposal of synergistic “anchor-capture.”

**Supplementary Information:**

The online version contains supplementary material available at 10.1007/s40820-023-01171-w.

## Graphical abstract

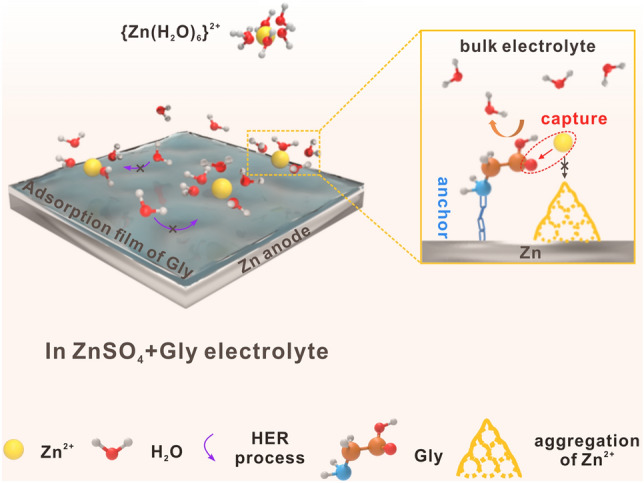

## Introduction

Rechargeable aqueous metal-ion batteries are of great potentials for future scale-up energy storage systems, on account of their low cost and high safety [1–4]. Among them, aqueous zinc-ion battery (AZIB) appears to be an attractive candidate due to the numerous intrinsic merits of Zn metal, *e.g.*, relatively low redox potential (− 0.76 V vs. SHE), high gravimetric capacity (820 mAh g^−1^) and volumetric capacity (5855 mAh cm^−3^), and abundant reserves [5–7]. Nonetheless, dendrite growth and side reactions caused by instability and complex chemistry on the Zn anode–electrolyte interface greatly decrease the utilization rate and reversibility, manifesting as low Coulombic efficiency (CE) [8–13].

The stability of Zn anode is largely determined by the diffusion and migration behavior of Zn^2+^ at the interface [14, 15]. In general, Zn^2+^ near the anode surface follows 2D diffusion mechanism, which aggregates at prior nucleation sites to capture electrons and then be reduced to form initial protrusion [16]. To minimize the surface energy, such reduction of Zn^2+^ tends to trigger the protrusion vertical growth and the formation of dendrites, leading to an increased surface area of Zn exposing to water molecules to accelerate the hydrogen evolution reaction (HER) [17]. The OH^−^ generated by hydrogen evolution elevates the local pH value near the anode and react with ZnSO_4_ electrolyte to further deteriorate the interface by generating the inactive by-product of Zn_4_SO_4_(OH)_6_·*x*H_2_O (ZSH) on the anode [18], which exacerbates the dendrite growth and side reactions.

Up till now, many progress has been achieved in surface modification [19–22], structural design [23, 24], and electrolyte modification [25–28] to address the above issues. Among them, electrolyte modification is one of the most effective strategies with wide applications. Natural biomolecules, such as amino acids and their derivatives, are attracting great attention for modifying the electrolytes. For instance, it was found that silk fibroin (SF) with mainly β-sheet conformation into ZnSO_4_ electrolyte can adhere to the surface of Zn anode and regulate the uniform Zn deposition [27, 29], while the one with the secondary structure transformation from α-helices to random coils in the aqueous electrolytes is inclined to participate in Zn^2+^ solvation structure due to more exposed polar groups [30]. Moreover, Xu et al. [31] proposed a lysozyme membrane constituted by β-configuration dominant amyloid aggregates to facilitate uniform Li^+^ flux in lithium metal batteries. As for amino acids, positively charged ones such as arginine are confirmed to electrostatically adsorb on Zn anode surface and regulate the interface charge states [32]. Cysteine, which possesses more complex configuration, contributing to the reconstruction of solvation structure and anode–electrolyte interface [33]. These additives are rich in various polar groups such as amino (−NH_2_), carboxyl (−COOH), sulfhydryl (−SH), etc. However, the efficacy of additives and the mechanism elucidation for specific polar group remain yet to be explored, which are especially critical for precisely designing functional additives applied into AZIBs.

Herein, the simplest amino acid, glycine (Gly), which consists of one amino and one carboxyl was selected as the electrolyte additive. The adsorption of amino promotes Gly with an anchoring effect on the surface of Zn metal, enabling a stable Zn anode–electrolyte interface and inhibiting the side reactions caused by water decomposition. In addition, Zn^2+^ is captured owing to the strong coordination with carboxyl, thus alleviating its disordered diffusion. Under this synergistic “anchor-capture” effect, side reactions and dendrite growth at the Zn anode–electrolyte interface are significantly suppressed, realizing a long-lifespan and stable Zn anode with excellent rate performance and long-term cycling stability.

## Experimental Procedures and Calculations

### Electrolytes Preparation and Cathode Material Synthesis

Zinc sulfate heptahydrate (ZnSO_4_·7H_2_O, AR) and Zn foil (99.999%, 150–250 μm thickness) were purchased from Sinopharm Chemical Reagent Co., Ltd. Sodium sulfate (Na_2_SO_4_, AR) was produced by Macklin. Glycine (Gly, 99.5–100.5%), N-acetylglycine (*Ac*-Gly, 99%) and glycinamide hydrochloride (Gly-NH_2_, 98%) were supplied by Aladdin. Electrolytes were prepared by adding 0.1 M (M: mol L^−1^) of Gly, *Ac*-Gly and Gly-NH_2_ into 2 M ZnSO_4_ solution. The MnO_2_ cathode was synthesized by a facile hydrothermal method. Typically, 0.474 g of KMnO_4_ and 2.718 g of MnSO_4_ were dissolved in 30 mL deionized water separately. Then mixed these two solutions quickly and stirred for 30 min until the solution turned purple-brown. Next, the mixed solution was transferred to an autoclave and heated at 140 °C for 12 h. The obtained precipitates were washed with deionized water by several times and dried in air at 80 °C for 12 h.

### Material Characterizations

The surface morphology and phase structures were assessed by the scanning electron microscopy (SEM, Hitachi S-4800) and X-ray diffraction analyzer (XRD, Bruker D8 diffractometer, Co-K*α*, *λ* = 1.789 Å). Fourier transform infrared spectroscopy (FTIR) was carried out on Thermo Scientific Nicolet iS20. X-ray photoelectron spectroscopy (XPS) was performed by Thermo Scientific K-Alpha equipped with Al K*α* X-ray source (12 kV, 6 mA). The O–H stretching of different electrolytes was collected by Raman spectroscopy (Raman, HORIBA Scientific LabRAM HR Evolution, 532 nm laser). The solvation structure of Zn^2+^ was confirmed by nuclear magnetic resonance spectrometer (^2^H NMR, Bruker 400 MHz), the solvent was D_2_O.

### Electrochemical Measurements

The Zn–Zn symmetric cells, Zn-Ti half cells and Zn-MnO_2_ full cells were assembled in atmosphere using CR2025 coin-type cell, which were tested by the Neware BTS-5 test system. Electrodes were cut into disks with a diameter of 16 mm with glass fiber filters (Whatman) used as separators. The cathode electrode was prepared by mixing MnO_2_, Ketjen black (KB) and polyvinylidene fluoride (PVDF) with a mass ratio of 7:2:1 in N-methyl-2-pyrrolidone (NMP) and casted onto Ti foils (10 μm). The electrode was dried at 120 °C for 12 h in vacuum. The mass loading of active material was around 2.5 mg.

The cyclic voltammetry (CV), linear sweep voltammetry (LSV), potentiodynamic scanning (Tafel), chronoamperometry (CA) measurements and electrochemical impedance spectroscopy (EIS) were carried out on the CHI760E electrochemical workstation. The average differential capacitance was derived from CV data with different scan rates (from 2 to 10 mV s^−1^) and a voltage range of − 15 to 15 mV. The hydrogen evolution performance was recorded by performing LSV test at 5 mV s^−1^ with Ti foil as the working electrode, Zn plate as the counter electrode and Ag/AgCl as the reference electrode, respectively. Tafel plots were performed at a scan rate of 0.01 V s^−1^ with Zn plate as the working electrode, Ti foil as the counter electrode and Ag/AgCl as the reference electrode. The CA curves were measured at a fixed overpotential of − 150 mV. The EIS measurement was finished with a frequency range of 0.01 ~ 1,000,000 Hz. The galvanostatic cycled Zn plates were obtained from two-electrode systems using Zn plates (1 cm $\times$ 3 cm) as both working and counter electrodes soaked in 2 M ZnSO_4_ and 2 M ZnSO_4_ + 0.1 M Gly/*Ac*-Gly/Gly-NH_2_ electrolytes. The soaking area of each electrode was 1 cm^2^.

The transfer number of Zn^2+^ was calculated according to potentiostatic polarization method [34], which can be given by:

1 $${t}_{{\mathrm{Zn}}^{2+}}= \frac{{I}_{\mathrm{SS}}\left(\Delta V-{I}_{0}{R}_{0}\right)}{{I}_{0}\left(\Delta V-{I}_{\mathrm{SS}}{R}_{\mathrm{SS}}\right)}$$

where ${I}_{\mathrm{SS}}$ and ${I}_{0}$ represent the steady-state current and initial current, ${R}_{\mathrm{SS}}$ and ${R}_{0}$ are the charge transfer resistances of steady-state and initial state, and $\Delta V$ is the applied overpotential (5 mV).

### Ab-initio Calculations

The surface adsorption calculation related to the interaction between Zn slab and molecules was performed by using the Vienna ab initio simulation package (VASP) [35, 36] based on Density functional theory (DFT). The projector augmented-wave (PAW) method [37] was carried out to represent the interactions of electrons with ion cores. The generalized gradient approximation (GGA) parameterized by the Perdew-Burke-Ernzerhof (PBE) method [38] with D3 correction [39] that was used to describe the van der Waals corrections. The energy cutoff was set to be 420 eV. The Brillouin-zone integration was sampled with a Γ-centered k-point mesh of 2 $\times$ 2 $\times$ 1 for all adsorption calculations. The convergence criteria for forces and energy were set to 0.02 eV Å^−1^ and 10^–5^ eV, respectively.

On the Z direction, there is 15 Å vacuum to avoid the interaction between adjacent images for slab model. A 5 $\times$ 5 supercell with four-layer Zn (002) slab was used to represent the absorbed surface for molecules, and the bottom two layers were fixed to simulate the bulk property. Visualization of the crystal structures was performed using VESTA [40] and data post-processing were used by VASPKIT code [41]. The adsorption energy between Zn slab and different molecules was defined as following equation:

2 $${E}_{\mathrm{ads}}={E}_{\mathrm{Zn}-\mathrm{Slab}+\mathrm{Molecules}}-{E}_{\mathrm{Zn}-\mathrm{Slab}}-{E}_{\mathrm{Molecules}}$$

where ${E}_{\mathrm{Zn}-\mathrm{Slab}+\mathrm{Molecules}}$, ${E}_{\mathrm{Zn}-\mathrm{Slab}}$ and ${E}_{\mathrm{Molecules}}$ represent the total energies of the Zn (002) Slab with adsorbed molecules, Zn (002) slab, and adsorbed molecules, respectively.

### Molecular Dynamics (MD) Simulations

Classical MD simulations were carried out using GROMACS 2022.2 [42] to provide insights on the electrolytes designed in this work. The force field parameters of Zn^2+^ ions and SPC/E [43] water model were obtained with Amber03 force field [44]. The GAFF force field [45] parameters of glycine and sulfate ion were generated with Acpype program [46], and the corresponding atom charges were based on restrained electrostatic potential (RESP) charges generated by Multiwfn [47]. Initially, 12 Gly, 40 Zn, 40 SO_4_ and 671 H_2_O molecules were packed into a 30 $\times$ 30 $\times$ 30 Å^3^ box using the packmol software [48].

For comparison, zinc sulfate solution was simulated by packing 40 Zn, 40 SO_4_ and 703 H_2_O molecules in a 30 $\times$ 30 $\times$ 30 Å^3^ box. All the systems were first submitted to energy minimization by using the steepest descent method. Then, they were heated from 10 to 298.15 K in 100 ps, followed by 200 ps equilibration under isothermal–isobaric ensemble (NPT) at 1 bar. For the production run, an additional 20 ns NPT simulation was performed. The integration time step was 1 fs. For NPT simulations, the temperature was controlled by coupling the system with a Nosé–Hoover thermostat [49] at a time constant of 2 ps, and the pressure was controlled using the Parrinello–Rahman pressure [50] coupled with a 5 ps time constant. Electrostatic interactions were treated using the Particle–Mesh–Ewald (PME) methods [51, 52] with a 1.3 nm cutoff distance. VMD software [53] was used to visualize the systems and obtain the ion association state.

### Quantum Chemistry (QC) Calculations

Quantum chemistry (QC) calculations were performed using the Gaussian 16 software [54] to calculate the structures and binding energies of Zn^2+^-Gly and Zn^2+^-H_2_O complexes. The structure optimization and frequency calculations were performed at B3LYP-D3(BJ)/def2-TZVP level [55, 56]. Then a single-point energy calculation of each optimized structure was performed at the same functional and basis set. The universal solvation model SMD [57] was used to simulate the aqueous environment. The binding energy (BE) is calculated as follows:

3 $${E}_{\mathrm{BE}}={E}_{\mathrm{AB}}-\left({E}_{\mathrm{A}}+{E}_{\mathrm{B}}\right)-{E}_{\mathrm{BSSE}}$$

where ${E}_{\mathrm{AB}}$, ${E}_{\mathrm{A}}$, and ${E}_{\mathrm{B}}$ denote the total energies of the AB complexes, bare A, and bare B, respectively. ${E}_{\mathrm{BSSE}}$ is the basis set superposition error (BSSE) correction energy [58], which is used to correct the energy of interaction in all the complexes.

## Results and Discussion

### “Anchor” Role of Amino Group for Anti-Corrosion

**Fig. 1:**
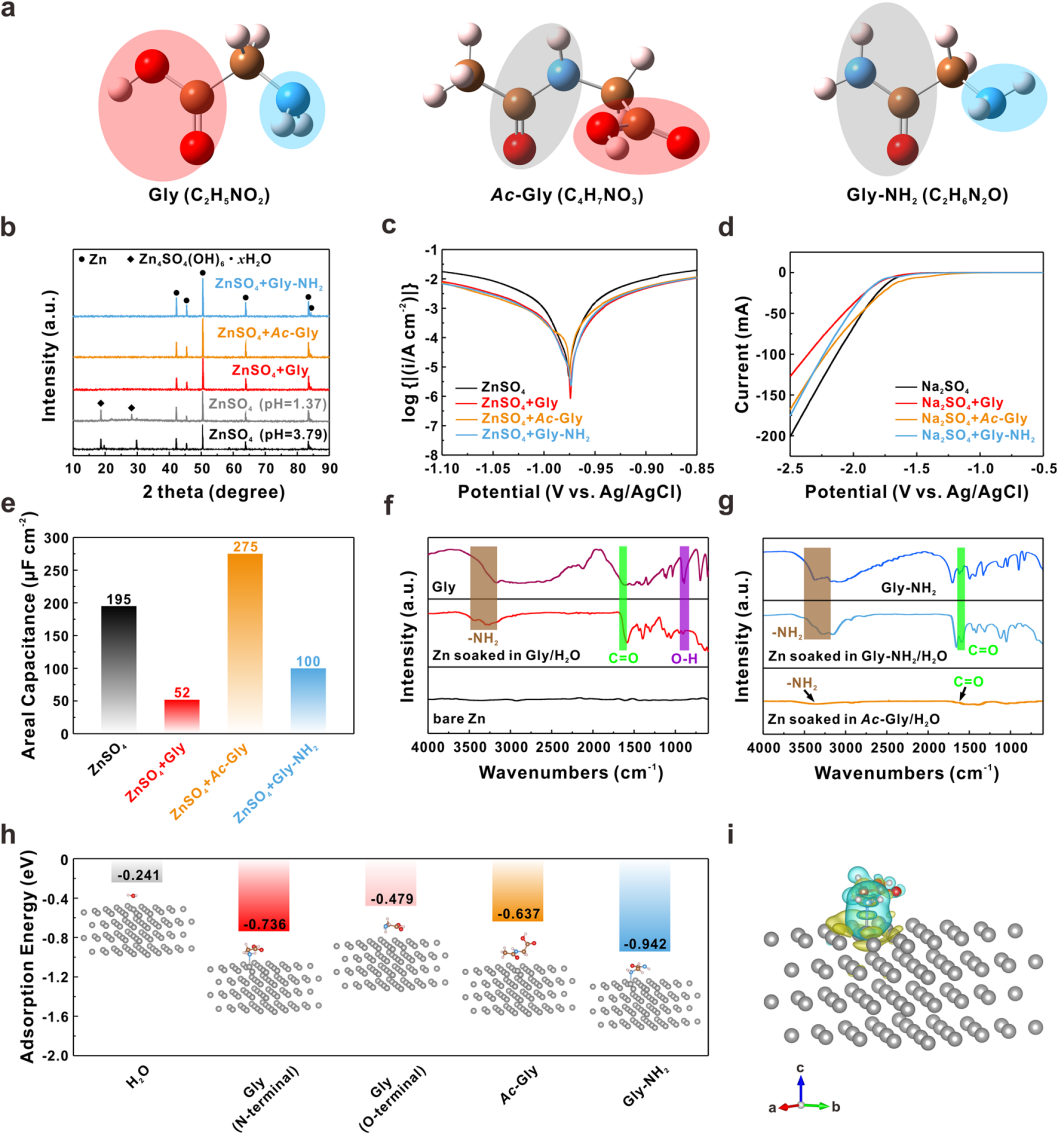
**a** Molecular structures of Gly, *Ac*-Gly and Gly-NH_2_. The blue, brown, red and pink balls represent N, C, O and H atoms, respectively. **b** XRD patterns of Zn plates soaked in different solutions for 7 days. The pH values of ZnSO_4_ + Gly/*Ac*-Gly/Gly-NH_2_ solutions were adjusted to 1.37. **c** Tafel plots of the Zn anode tested with three-electrode system in different electrolytes. **d** LSV curves of Zn plate tested with three-electrode system in different electrolytes. **e** Average differential capacitance for Zn in different electrolytes. FTIR spectra of Zn plates soaked in different solutions for 7 days: **f** Gly/H_2_O solution; **g** *Ac*-Gly/H_2_O and Gly-NH_2_/H_2_O solutions. **h** Adsorption energies comparison of H_2_O, Gly, *Ac*-Gly and Gly-NH_2_ molecules on the Zn (002) facet, insets are the corresponding adsorbed models for different situations. The gray balls represent Zn atoms. **i** The charge density difference of the Zn slab with Gly molecule along the c axis (iso-value = 6 $\times$ 10^–4^ e Bohr^−3^). The yellow and cyan iso-surfaces represent the increase and decrease in electron density, respectively

First of all, 2 M ZnSO_4_ electrolytes with different concentrations of Gly (0.05/0.1/0.5 M) were used to assemble Zn–Zn symmetric cells. Under the galvanostatic condition of 1 mA cm^−2^ and 1 mAh cm^−2^, the cell with 0.1 M Gly shows an ultra-stable cycling life over 1000 h (Fig. S1). Therefore, the concentration of 0.1 M was chosen for further study on Gly. To accurately compare the roles of different polar groups, N-acetylglycine (*Ac*-Gly) and glycinamide hydrochloride (Gly-NH_2_) are selected as the other two additives, which are derived by the amidation of amino and carboxyl groups in Gly, respectively. The detailed molecular structures of Gly, *Ac*-Gly and Gly-NH_2_ are shown in Fig. 1a. The amide group (–CO–NH–, highlighted by light gray translucent oval area) is usually less active [59], which has negligible effect on Zn anode during cycling. After adding 0.1 M Gly/*Ac*-Gly/Gly-NH_2_ into 2 M ZnSO_4_ solution, the pH values of electrolytes were first determined. As can be seen from Fig. S2, the pH value declines from 3.79 for bare ZnSO_4_ to 2.94/1.37/2.52 for ZnSO_4_ + Gly/*Ac*-Gly/Gly-NH_2_, respectively. To evade the effect of pH, dilute sulfuric acid (10 wt.%) was added to the ZnSO_4_ and ZnSO_4_ + Gly/Gly-NH_2_ electrolytes to adjust the pH to 1.37.

Compared with the normal ZnSO_4_ solution (pH = 3.79), after immersed in ZnSO_4_ solution with lower pH for 7 days, the Zn plate presents the similar morphology with rough surface covered by a mass of irregular flake-like by-products (Fig. S3), which are confirmed by the X-ray diffraction (XRD) patterns as Zn_4_SO_4_(OH)_6_·*x*H_2_O (Fig. 1b), indicating that the decline in pH would not inhibit the formation of by-products. On the contrary, no undesired by-products and diffraction peaks appear for the Zn plate immersed in ZnSO_4_ + Gly/Gly-NH_2_ solutions (pH = 1.37), as well as the overall smooth surfaces display step-like or isolated Zn islands randomly distributed (highlighted by yellow solid line circles) morphology, respectively. Under the presence of *Ac*-Gly, large cavities (highlighted by yellow dash line circles) and a rugged morphology are discovered on the surface despite no by-products to be observed. Moreover, the pH values of these solutions following immersion with Zn plates were also determined. As shown in Fig. S4, the pH values of ZnSO_4_ + Gly/*Ac*-Gly/Gly-NH_2_ solutions vary smaller than that of ZnSO_4_ solution, implying the better anti-corrosion property of these additives. Especially, the increase of pH for ZnSO_4_ + Gly solution is only 0.65, further demonstrating that Gly molecules can effectively inhibit side reactions and stabilize the local pH.

To assess the corrosion behaviors of Zn anode, the Tafel plots of Zn–Zn symmetric cells using different electrolytes are recorded in Fig. 1c, with corresponding corrosion current density (${j}_{\mathrm{cor}}$)/corrosion potential (${V}_{\mathrm{cor}}$) summarized in Table S1. The difference between each ${V}_{\mathrm{cor}}$ is negligible. As expected, the Zn anode in ZnSO_4_ + Gly electrolyte delivers the lowest ${j}_{\mathrm{cor}}$ of 1.11 mA cm^−2^, which indicates the prominent anti-corrosion. The decreases of ${j}_{\mathrm{cor}}$ compared to bare ZnSO_4_ electrolyte with the addition of *Ac*-Gly and Gly-NH_2_ also demonstrate that they suppress the corrosion of Zn anode to some extent. Moreover, the HER performance is a critical indicator to evaluate the stability of Zn anode, which is reflected by the magnitude of cathodic current in linear sweep voltammetry (LSV) curves. To alleviate the electrochemical reduction of Zn^2+^ on surface, the base electrolyte is changed from 2 M ZnSO_4_ to 1 M Na_2_SO_4_ solution. In Fig. 1d, the increased HER overpotential and decreased HER current further confirm the positive roles of Gly and Gly-NH_2_ in blocking side reactions. However, only decreased HER current can be observed in Na_2_SO_4_ + *Ac*-Gly electrolyte, which can be attributed to the de-solvation effect of carboxyl (as discussed in the following section).

In order to understand the anti-corrosion performance of Zn anode in different electrolytes, the differential capacitance tests were performed. The average differential capacitance ($\overline{C}$) is closely relevant to the electric double-layer (EDL) structure near the electrode surface [60–62], which can be derived from the following equation:

4 $$\overline{i}= \overline{C }\cdot v$$

where $\overline{i}$ is the average current density and $v$ is the corresponding scan rate of a cyclic voltammetry (CV) curve.

As shown in Figs. S5 and 1e, the $\overline{C}$ of Zn anode significantly decreases to 52 and 100 μF cm^−2^ with Gly and Gly-NH_2_ additives, respectively. We conjecture that both Gly and Gly-NH_2_ can participate into the EDL structure and interact with Zn anode surface. In contrast, after adding *Ac*-Gly, a prominent increase of $\overline{C}$ is observed from 195 to 275 μF cm^−2^. This might be caused by the corrosion of Zn anode that leads to more exposure of surface area to electrolyte, as large cavities observed in SEM image in Fig. S3. To verify the hypothesis mentioned above, the Fourier transform infrared spectroscopy (FTIR) was performed. The soaked Zn plates from Gly/H_2_O and Gly-NH_2_/H_2_O solutions exhibit the similar spectra variation compared with bare Zn in the range of 600 ~ 1700 cm^−1^, including the C=O stretching at 1597 cm^−1^ and O–H bending at 902 cm^−1^, respectively (Figs. 1f, g). In addition, peaks at 3200 ~ 3500 cm^−1^ are observed which correspond to the -NH_2_ stretching.

These results convincingly confirm that Gly and Gly-NH_2_ can spontaneously adsorb on Zn surface in solution media. Nevertheless, for Zn plate soaked in *Ac*-Gly/H_2_O solution, weak signals of –NH_2_ and C=O stretching are detected, indicating the poor adsorption of *Ac*-Gly (Fig. 1g). The energy-dispersive X-ray spectroscopy (EDS) mapping also verifies the uniform coverage of Gly on Zn surface (Fig. S6). Ab-initio calculations were also conducted to study the adsorption energies (${E}_{\mathrm{ads}}$) of different molecules on the Zn (002) facet (Fig. 1h). Interestingly, the interaction between Zn surface and Gly molecule is much stronger than that of H_2_O molecule (− 0.241 eV), no matter the N-terminal from amino or the O-terminal in –C=O from carboxyl. Specifically, Gly molecule prefers to adsorb on Zn surface by amino since the ${E}_{\mathrm{ads}}$ in N-terminal state is much larger than that in O-terminal state (− 0.736 vs. − 0.479 eV). The relatively larger ${E}_{\mathrm{ads}}$ of Gly-NH_2_ (− 0.942 eV) and smaller ${E}_{\mathrm{ads}}$ of *Ac*-Gly (− 0.637 eV) on Zn surface also demonstrate the prior interplay between amino and Zn atoms.

To determine whether the amino is physically or chemically adsorbed on Zn surface, the charge density difference calculations and high-resolution X-ray photoelectron spectroscopy (XPS) spectra were carried out. In Fig. 1i, apparent overlapped electron cloud between Zn and N atoms is found, suggesting a stable affinity between Gly and Zn. Meanwhile, the characteristic peak of N–Zn is distinctly observed at 398.9 eV in the N 1*s* spectrum (Fig. S7). These results confirm that Gly and Gly-NH_2_ chemically anchor on the Zn surface via amino group, which plays a dominant role in inhibiting side reactions.

### “Capture” Role of Carboxyl Group for Uniform Deposition

**Fig. 2:**
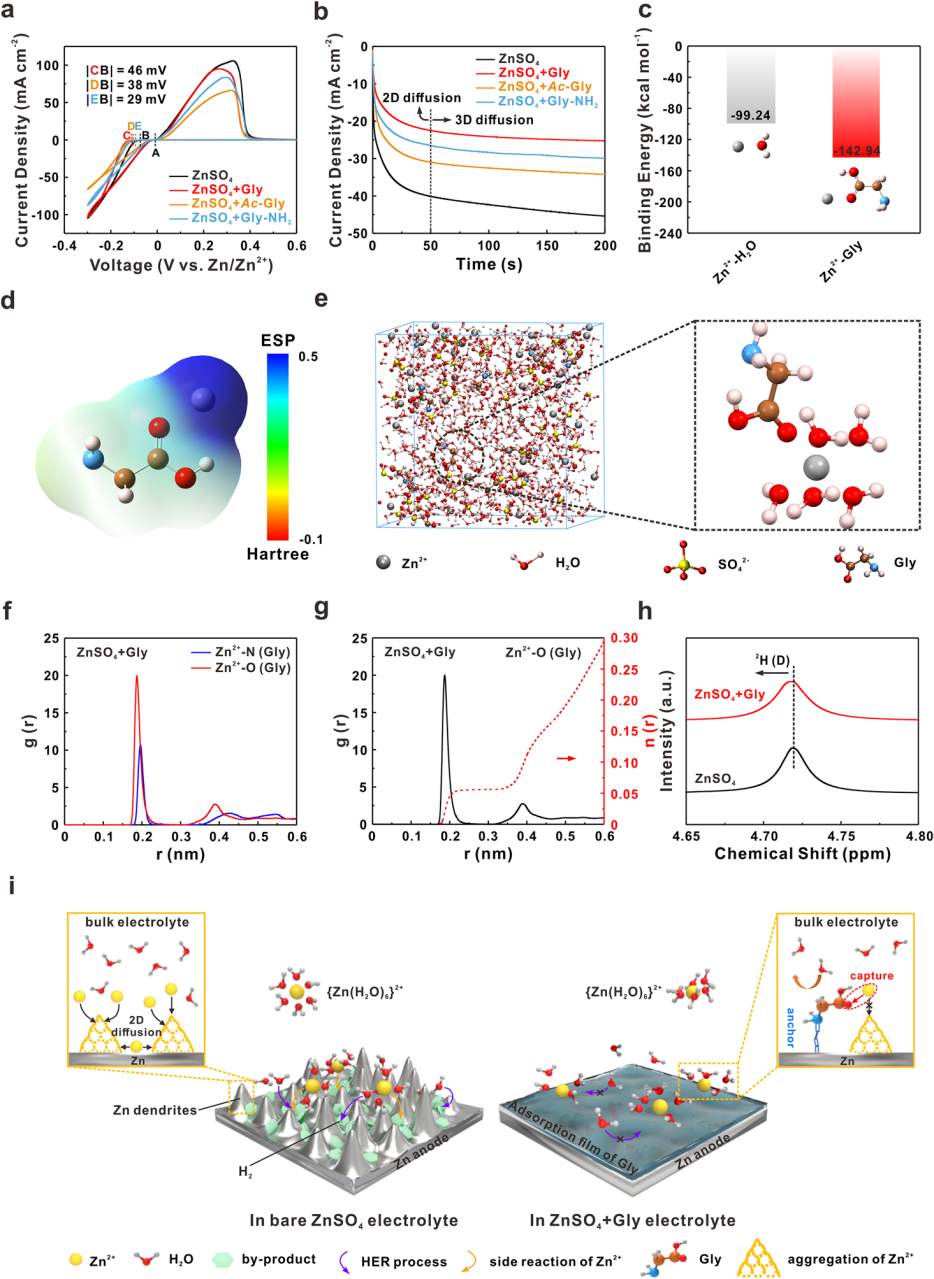
**a** CV curves for Zn nucleation in Zn-Ti cells using bare ZnSO_4_ and ZnSO_4_ + Gly/*Ac*-Gly/Gly-NH_2_ electrolytes. **b** The CA curves of Zn anode tested in Zn–Zn symmetric cells using different electrolytes. **c** Binding energies for Zn^2+^ with different molecules (H_2_O and Gly) under QC calculations. **d** Electrostatic potential mapping of the Gly-Zn^2+^ compound. **e** 3D snapshot of MD simulations for ZnSO_4_ + Gly electrolyte and partial enlarged snapshot representing Zn^2+^ solvation structure. **f** Simulated radial distribution functions (RDFs) for Zn^2+^-N (Gly) and Zn^2+^-O (Gly) collected from MD simulations in ZnSO_4_ + Gly electrolyte. **g** Simulated RDFs and coordination numbers analysis for Zn^2+^-O (Gly) in ZnSO_4_ + Gly electrolyte. **h** NMR spectra for ZnSO_4_/ZnSO_4_ + Gly electrolytes. **i** Schematic illustration of Zn deposition behaviors in ZnSO_4_ electrolyte with/without Gly

Apart from the inhibition of side reactions, the effect of Gly, *Ac*-Gly and Gly-NH_2_ on Zn^2+^ deposition behavior was also explored. As shown in Fig. 2a, compared with bare ZnSO_4_ electrolyte, the nucleation overpotentials of Zn^2+^ in ZnSO_4_ + Gly and ZnSO_4_ + *Ac*-Gly electrolytes significantly increase by 46 and 38 mV, respectively, much larger than that in Gly-NH_2_-containing electrolyte (29 mV). It is well known that a higher nucleation energy barrier for Zn^2+^ is conducive to the formation of finer nuclei and uniform deposition [32, 63, 64]. Subsequently, the chronoamperometry (CA) measurements were utilized to assess the evolution of deposition and growth of Zn nuclei under an overpotential of − 150 mV with Zn–Zn symmetric cells (Fig. 2b). The growth rates of current density caused by polarization during 50–200 s were calculated and shown in Table S2. In general, the rapid increase of current density with time in bare ZnSO_4_ electrolyte reflects that the surface of Zn anode is dominated by 2D diffusion mode, in which Zn^2+^ migrates along the Zn surface and the consequent “tip effect” leads to the continuous accumulation of Zn nuclei, which brings about the severe dendrite issues [16, 65, 66].

In contrast, the addition of *Ac*-Gly and Gly-NH_2_ can reduce the current growth rate, which is caused by the restricted 2D diffusion of Zn^2+^. For Gly-containing electrolyte, the disordered Zn^2+^ flux is regulated and Zn^2+^ is directly reduced on the surface, yielding a stable 3D diffusion mode with the lowest current density response. The transfer number of Zn^2+^ (${t}_{{\mathrm{Zn}}^{2+}}$) in different electrolytes was tested by potentiostatic polarization method. Figures S8a and c show the electrochemical impedance spectroscopy (EIS) spectra of Zn–Zn symmetric cells in bare ZnSO_4_ and ZnSO_4_ + Gly electrolytes and the corresponding CA curves under the applied overpotential of 5 mV are displayed in Figs. S8b, d. As evidenced in Fig. S8e, the symmetric cell delivers a higher ${t}_{{\mathrm{Zn}}^{2+}}$ with Gly-containing electrolyte, further indicating the more homogeneous Zn^2+^ flux and concentration gradient at the interface [67].

The above results have proved that the additives can regulate the kinetics of Zn^2+^. Here, the interactions among Zn^2+^, H_2_O and Gly molecules were further analyzed using quantum chemistry (QC) calculations. The calculated binding energies suggest that Zn^2+^ appears to be more favored to combine with Gly than H_2_O (Fig. 2c). Besides, the electrostatic potential value for Gly molecule significantly increases when Zn^2+^ approaches the carboxyl group (Figs. 2d and S9), implying the transfer of electrons from nucleophilic site (carboxyl group) to Zn^2+^, which in favor of building firm bond among them in an implicit solution environment. Molecular dynamics (MD) simulations were carried out to investigate the solvation structure of Zn^2+^ in ZnSO_4_ + Gly electrolyte. In Fig. 2e, the double bonded oxygen atom from carboxyl in one Gly molecule obviously participates into the solvation sheath and replaces one of the H_2_O molecules around Zn^2+^, indicating an explicit change in solvation structure.

The corresponding radial distribution functions (RDFs) and coordination number analysis in different electrolytes were also performed. In bare ZnSO_4_ electrolyte, a sharp peak of Zn^2+^–O at around 1.9 Å away from Zn^2+^ is observed, which should refer to the participation of H_2_O into the solvation sheath, and the average coordination number is simulated to be 5.5 (Fig. S10). In ZnSO_4_ + Gly electrolyte, it is clear to see that the peak of Zn^2+^–O (Gly) appears at a closer distance and displays much stronger intensity than that of Zn^2+^-N (Gly), and the average coordination number of Zn^2+^–O (H_2_O) is reduced to be 5.3, demonstrating that Gly molecule mainly coordinates with Zn^2+^ via the carboxyl group rather than the amino group and can partially de-solvate Zn^2+^ with a new solvation structure (Figs. 2f, g and S11). For further verifying the de-solvation effect of carboxyl group, the Raman spectra and nuclear magnetic resonance (NMR) spectra of different electrolytes were conducted. As shown in Fig. S12a, b, the O–H stretching vibration at around 3100 ~ 3680 cm^−1^ shifts to a higher wavenumber after adding Gly and *Ac*-Gly, demonstrating that both the molecules with carboxyl group have stronger interaction with water molecules and could break the original hydrogen bonding network, which weaken the activity of water [68]. On the contrary, the Gly-NH_2_ molecules without carboxyl group do not cause shift of the O–H stretching compared with bare ZnSO_4_ (Fig. S12c). As for the NMR results in Fig. 2h, the ^2^H peak of D_2_O shifts from 4.719 to 4.716 ppm in ZnSO_4_ + Gly electrolyte, implying that more free water is released, which further proves that Gly can change the solvation structure of Zn^2+^ [69]. Furthermore, the mean-squared displacement (MSD) versus time was performed to characterize the diffusion rate of Zn^2+^ in different electrolytes (Fig. S13). By adding Gly, the plot slope for MSD versus time increases, which can be inferred that Zn^2+^ diffuses faster to Zn surface in ZnSO_4_ + Gly electrolyte.

### Synergistic “Anchor-Capture” Mechanism

Based on the above results, a synergistic “anchor-capture” effect (Fig. 2i) of amino and carboxyl groups in Gly molecule emerges as the mechanism of stabilizing Zn anode. In details, Zn^2+^ would solvate with H_2_O to form {Zn(H_2_O)_6_}^2+^ in bare ZnSO_4_ electrolyte and migrate to the surface of Zn anode under the internal electric field. On the one hand, the H_2_O molecule would decompose and generate H_2_ after contacts with Zn metal, thus elevating the pH value of local regions and further promoting the formation of by-products (ZSH). On the other hand, Zn^2+^ randomly diffuses along the rough metal surface and aggregates at the prior nucleation sites to form small prominence. The consecutively deteriorated surface conditions significantly induce the growth of Zn dendrites, eventually leading to the failure of battery system by piercing the separator and causing short circuit.

On the contrary, the introduction of Gly molecule can firmly anchor on the Zn anode surface via the N atom of amino, which prevents the contact between H_2_O and Zn metal, thus protecting Zn anode from side reactions. Meanwhile, the Zn^2+^ is captured by Gly due to the strong nucleophilicity of carboxyl, effectively confining the disordered 2D diffusion and guiding the uniform deposition. At the case of Gly-NH_2_ and *Ac*-Gly, amino or carboxyl groups alone cannot effectively stabilize the interface, thus eventually Zn anode would deteriorate and come to failure.

### Synergistic “Anchor-Capture” Enabled Electrochemical Performance

**Fig. 3:**
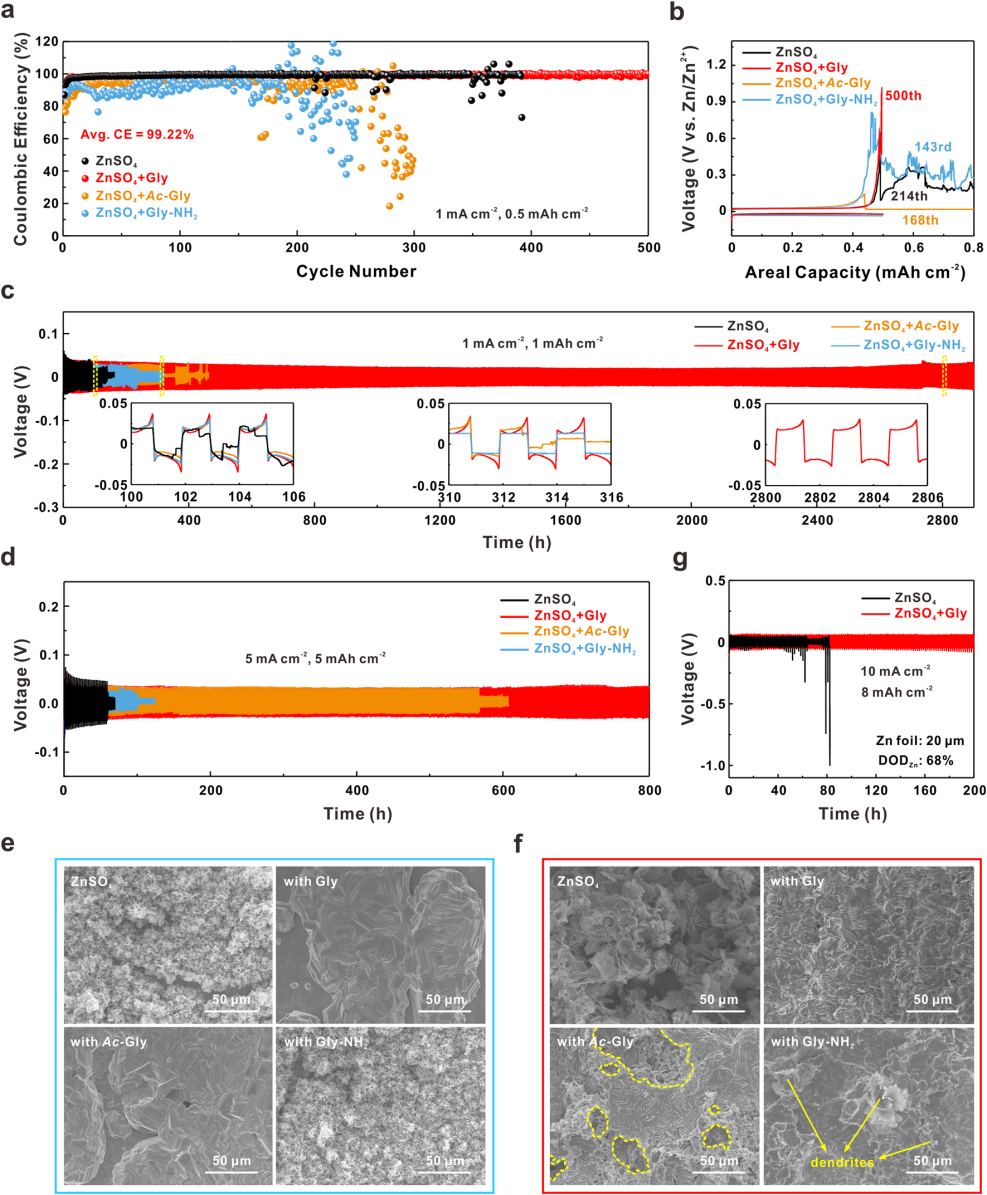
**a** CE comparison of Zn-Ti cells in bare ZnSO_4_ and ZnSO_4_ + Gly/*Ac*-Gly/Gly-NH_2_ electrolytes at 1 mA cm^−2^ and 0.5 mAh cm^−2^ and **b** corresponding voltage profiles at different cycles, respectively. The voltage profiles of Zn–Zn symmetric cells using different electrolytes performed at **c** the current density of 1 mA cm^−2^ with the areal capacity of 1 mAh cm^−2^, the insets are detailed voltage profiles at different cycle time ranges; **d** the current density of 5 mA cm^−2^ with the areal capacity of 5 mAh cm^−2^. **e** The SEM images of Zn electrodeposition on Zn anodes in different electrolytes for 1 h at 5 mA cm^−2^. **f** Surface morphologies of Zn anode after 50 cycles in different electrolytes under the condition of 5 mA cm^−2^ and 5 mAh cm^−2^. **g** The voltage profiles of Zn-Zn symmetric cells using ZnSO_4_ and ZnSO_4_ + Gly electrolytes performed at the current density of 10 mA cm^−2^ with the areal capacity of 8 mAh cm^−2^ (rolled Zn foil as anode with the DOD_Zn_ of 68%)

First, the long-term CE performances in different electrolytes are compared by assembling Zn-Ti asymmetric cells. As shown in Figs. 3a and b, the introduction of Gly achieves an ultra-stable operation over 500 cycles and delivers a high average CE of 99.22% at 1 mA cm^−2^ and 0.5 mAh cm^−2^, which illustrate that the Zn anode can maintain high reversibility under the synergistic effect of amino and carboxyl groups. For the bare ZnSO_4_ electrolyte, the Zn-Ti cell displays a notable fluctuating CE at about the 214th cycle, and the cut-off voltage of charging (1.0 V) cannot be reached, primarily due to the deteriorating dendrite growth and severe side reactions. However, Zn-Ti cells with *Ac*-Gly and Gly-NH_2_ exhibit more chaotic fluctuation of CE, mainly ascribed to the uneven stripping and the concentrated plating beyond cavities or near dendrites. Even under a much faster kinetics at 10 mA cm^−2^ and higher deposition capacity of 1 mAh cm^−2^, the Zn anode with Gly can still keep stable cycling with a high average CE of 99.15% for 580 cycles, which significantly surpasses that of *Ac*-Gly and Gly-NH_2_ (Fig. S14). These results demonstrate the effectiveness of synergistic “anchor-capture” mechanism in homogenizing Zn deposition and promoting the reversibility of Zn anode.

The Zn–Zn symmetric cells using ZnSO_4_ + Gly electrolyte can also deliver much better cycling stability under galvanostatic condition. The voltage profiles in Fig. 3c depict that the Zn–Zn cell with Gly additive exhibits an ultra-long cycle lifespan over 2800 h without obvious overpotential fluctuation at a current density of 1 mA cm^−2^. On the contrary, the cell cycled in ZnSO_4_ electrolyte maintains stable for merely 90 h and fails with a sudden voltage drop, as a result of the short circuit due to the formation of disordered Zn dendrites which penetrate through the separator [70]. The stability of Zn anode last only for 190 and 310 h with amino (Gly-NH_2_) or carboxyl (*Ac*-Gly) groups alone, respectively. Moreover, as shown in EIS results of Fig. S15, the Zn–Zn cell with Gly-containing electrolyte displays a similar charge transfer resistance (${R}_{\mathrm{ct}}$) compared with that in bare ZnSO_4_ electrolyte before cycling, indicating that the adsorption of Gly has no negative impact on the charge transfer process near Zn anode surface. Nevertheless, the symmetric cell with Gly-containing electrolyte shows a lower ${R}_{\mathrm{ct}}$ than that with ZnSO_4_ electrolyte after 20 cycles, due to the robust anode–electrolyte interface constructed by the assistance of amino and carboxyl groups in Gly.

When the current density is increased to 5 mA cm^−2^, the symmetric cell with Gly also achieves an operation time of up to 800 h under the areal capacity of 5 mAh cm^−2^, which is about 16 times of that in bare ZnSO_4_ electrolyte (53 h, Fig. 3d). Interestingly, due to the regulated Zn^2+^ flux enabled by carboxyl group, the addition of *Ac*-Gly realizes much longer cycle life and higher cycle stability than that of Gly-NH_2_ in Zn–Zn symmetric cells, which is consistent with the results analyzed before. To evaluate the evolution of Zn deposition process, the electrodeposition of Zn^2+^ at 5 mA cm^−2^ on Zn anode was conducted and characterized by SEM. As shown in Fig. 3e, after 1 h of electrodeposition, the deposits are smooth and dense for ZnSO_4_ + Gly/*Ac*-Gly, whereas loose and full of dendrites for bare ZnSO_4_ and ZnSO_4_ + Gly-NH_2_. For longer time of electrodeposition (2 h, Fig. S16), unlike the highly dense and flat deposits in ZnSO_4_ + Gly electrolyte, there are some large pores generated with the presence of *Ac*-Gly, which may cause the failure of batteries during the subsequent cycling. The SEM images and XRD patterns of cycled Zn anodes in Figs. 3f and S17 also confirm the critical role of combined amino and carboxyl on stabilizing Zn anode. Even at a larger current density of 10 mA cm^−2^, Gly can ensure a superior operation stability of 175 cycles (350 h) with no fluctuation of voltage (Fig. S18).

The depth of discharge of Zn anode (DOD_Zn_) is one of the crucial criteria to evaluate the gravimetric energy density of ZIBs, referring to the fraction of Zn involved in the electrochemical redox reactions in Zn anode [71]. Since the commercial Zn foil (with a thickness slightly over 100 μm) used as anode at a low areal capacity is far too excessive, the utilization rate of Zn anode is extremely low (DOD_Zn_ $<$ 1%). In order to evaluate the stability of Zn anode at a higher DOD_Zn_, the thickness of commercial Zn foil was reduced by repeated rolling with several times. As shown in Fig. S19, the texture of rolled Zn foil is in accordance with that of commercial Zn foil, with a thickness of about 20 μm.

Subsequently, the voltage profiles of symmetric cells assembled with rolled Zn foils at different DOD_Zn_ were depicted in Figs. S20 and 3 g. At a DOD_Zn_ of 43% (the areal capacity is 5 mAh cm^−2^), a steady cycle lifespan for 400 h with less than 50 mV overpotential is achieved with the addition of Gly, whereas the cell with bare ZnSO_4_ electrolyte presents short circuit within 100 h. Even at a higher DOD_Zn_ of 68% (the areal capacity is 8 mAh cm^−2^), the Zn–Zn cell with Gly delivers a remarkable cycle stability up to 200 h. In contrast, large voltage polarization after 50 h is observed in the bare ZnSO_4_ electrolyte and the cell finally becomes open-circuited at 83 h. These results are strong proofs for ensuring AZIBs achieve practical application target (DOD_Zn_ $>$ 40%) with the synergistic effect of amino and carboxyl groups.

### Validation of Stable Anode–Electrolyte Interface Enhanced Full Cells with MnO_2_ Cathode

**Fig. 4:**
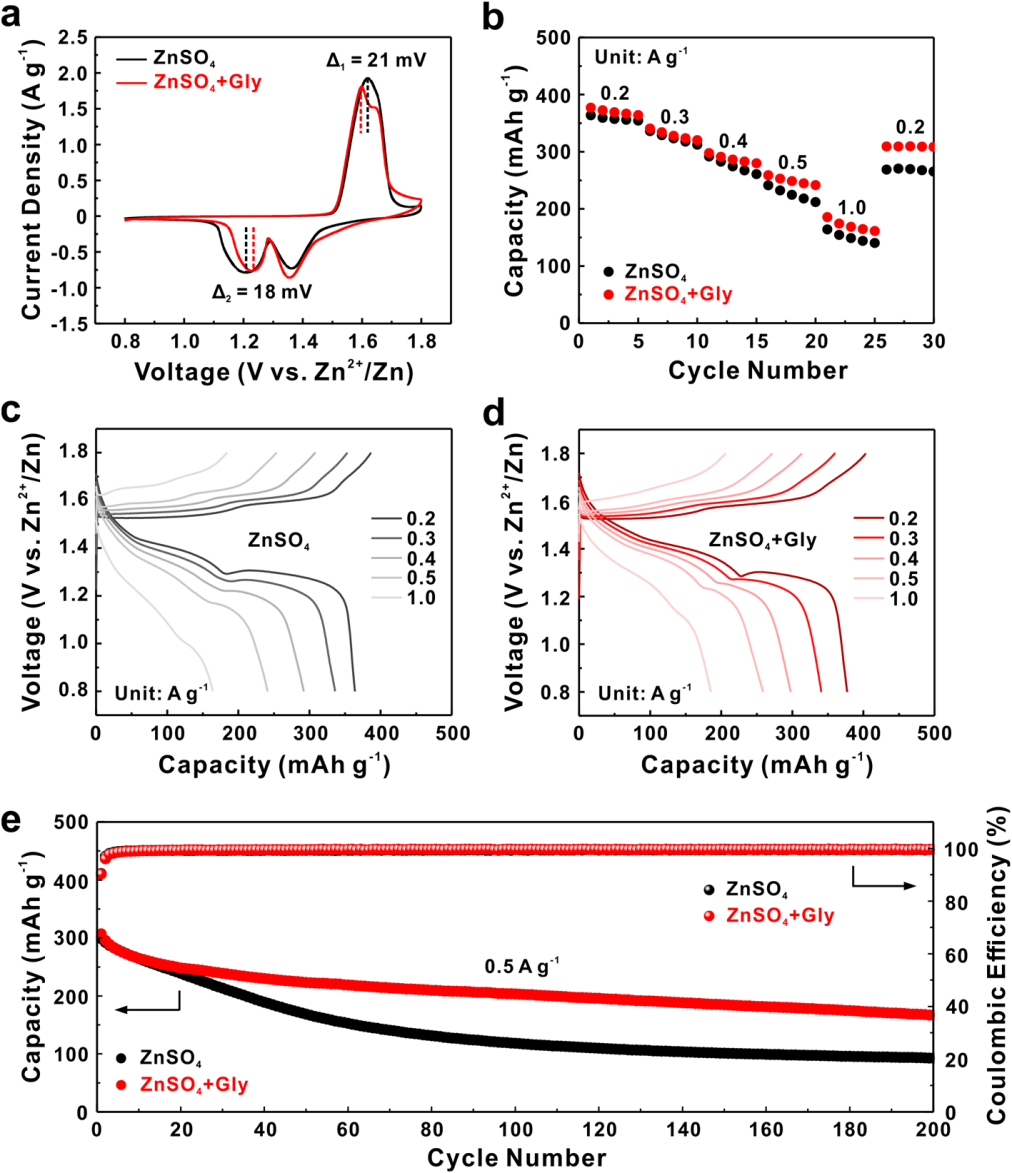
**a** CV curves of Zn-MnO_2_ full cells using ZnSO_4_ and ZnSO_4_ + Gly electrolytes. **b** Rate capability of Zn-MnO_2_ full cells at various current densities from 0.2 to 1.0 A g^−1^ in different electrolytes and **c**, **d** corresponding charge/discharge profiles. **e** Cycling performance of Zn-MnO_2_ full cells at the current density of 0.5 A g^−1^ with/without Gly. 0.1 M MnSO_4_ was added into each electrolyte

Accordingly, The Zn–MnO_2_ full cells were assembled with β-MnO_2_ cathode to further demonstrate the stable anode–electrolyte Interface enhanced AZIBs. The β-MnO_2_ powder synthesized by hydrothermal method possesses fine needle-like morphology with an average length of 2 μm and excellent crystallinity (Fig. S21). Notably, 0.1 M MnSO_4_ was added into both bare ZnSO_4_ and ZnSO_4_ + Gly electrolytes to alleviate the manganese dissolution during cycling [72]. Figure 4a shows the CV curves of Zn-MnO_2_ full cells with/without Gly. The similar redox peaks of both two curves suggest that Gly has no impact on the reaction mechanism of MnO_2_, which is supposed to be the H^+^ and Zn^2+^ co-insertion mechanism [73]. Obviously, the anodic peak shifts to lower voltage (Δ_1_ = 21 mV) and the cathodic peak shifts to higher voltage (Δ_2_ = 18 mV) in ZnSO_4_ + Gly electrolyte, demonstrating the diminished polarization and boosted reaction kinetics of MnO_2_ cathode material. Under the synergistic “anchor-capture” effect, the Zn-MnO_2_ full cell delivers a superior rate capability to that with bare ZnSO_4_ electrolyte (Fig. 4b). After cycling from 0.2 to 1.0 A g^−1^ (5 cycles for each current density), the Zn-MnO_2_ cell with Gly remains a high specific capacity of 309.4 mAh g^−1^ and preferable capacity retention of 82% when the current density returns to 0.2 A g^−1^, much higher than that in bare ZnSO_4_ electrolyte (269 mAh g^−1^, 73% of the initial capacity).

The charge–discharge profiles presented in Figs. 4c, d unveil that both two voltage plateaus during discharge process show better repeatability with increasing current density, further revealing the positive role of Gly on reaction reversibility of MnO_2_ cathode. Finally, the long-term cycling performance of Zn-MnO_2_ full cells with/without Gly was tested. In Fig. 4e, it is clear to see that the cell maintains a flat and steady cycle performance for 200 cycles with a higher discharge capacity of 167.2 mAh g^−1^ at 0.5 A g^−1^ in the presence of Gly, while a quick loss of capacity within 80 cycles is observed for the cell using bare ZnSO_4_ electrolyte, which delivers an inferior discharge capacity of 93 mAh g^−1^ (31% of the initial capacity) at 200th cycle. Furthermore, the specific capacity with bare ZnSO_4_ electrolyte rapidly decays from 105.1 to 16.7 mAh g^−1^ within 200 cycles at a current density of 3 A g^−1^ (Fig. S22a). In comparison, the cell with Gly additive delivers higher discharge capacity over the whole 200 cycles. Even at a higher current density of 5 A g^−1^, the Zn-MnO_2_ full cell assembled with Gly electrolyte exhibits a capacity retention of 75% (58.4 vs. 77.8 mAh g^−1^) after 200 cycles (Fig. S22b).

## Conclusions

In this work, Gly was used as an electrolyte additive to stabilize the Zn anode interface. Benefited from the synergistic “anchor-capture” effect of amino and carboxyl, Gly can steadily anchor on the Zn anode surface and capture the Zn^2+^ at the interface, therefore inhibiting HER process and the formation of by-products, which ensures uniformly deposition of Zn^2+^. This strategy achieves a stable long cycle life over 2800 h (at the condition of 1 mA cm^−2^ and 1 mAh cm^−2^) and high Zn utilization rate of 68% (areal capacity of 8 mAh cm^−2^) in Zn–Zn symmetric cells. Moreover, the reversibility of Zn stripping/plating process is significantly improved with a high average CE of 99.22%. Finally, the superior rate performance and long-term cycling stability of Zn–MnO_2_ full cells further verify the potential of Gly in practical application. This work would bring new insight into the selection and design of additives for promising AZIBs in large-scale energy storage.

### Supplementary Information

Below is the link to the electronic supplementary material.

Supplementary file1 (PDF 1454 KB)

## References

[1] Blanc LE, Kundu D, Nazar LF (2020). Scientific challenges for the implementation of Zn-ion batteries. Joule.

[2] Ma L, Schroeder MA, Borodin O, Pollard TP, Ding MS (2020). Realizing high zinc reversibility in rechargeable batteries. Nat. Energy.

[3] Zhang H, Liu X, Li H, Hasa I, Passerini S (2021). Challenges and strategies for high-energy aqueous electrolyte rechargeable batteries. Angew. Chem. Int. Ed..

[4] Zhang N, Chen X, Yu M, Niu Z, Cheng F (2020). Materials chemistry for rechargeable zinc-ion batteries. Chem. Soc. Rev..

[5] Jia X, Liu C, Neale ZG, Yang J, Cao G (2020). Active materials for aqueous zinc ion batteries: synthesis, crystal structure, morphology, and electrochemistry. Chem. Rev..

[6] Wang H, Tan R, Yang Z, Feng Y, Duan X (2021). Stabilization perspective on metal anodes for aqueous batteries. Adv. Energy Mater..

[7] Yang J, Yin B, Sun Y, Pan H, Sun W (2022). Zinc anode for mild aqueous zinc-ion batteries: challenges, strategies, and perspectives. Nano-Micro Lett..

[8] Cao Z, Zhuang P, Zhang X, Ye M, Shen J (2020). Strategies for dendrite-free anode in aqueous rechargeable zinc ion batteries. Adv. Energy Mater..

[9] Chao D, Zhou W, Ye C, Zhang Q, Chen Y (2019). An electrolytic Zn–MnO_2_ battery for high-voltage and scalable energy storage. Angew. Chem. Int. Ed..

[10] Han C, Li W, Liu HK, Dou S, Wang J (2020). Principals and strategies for constructing a highly reversible zinc metal anode in aqueous batteries. Nano Energy.

[11] Li TC, Fang D, Zhang J, Pam ME, Leong ZY (2021). Recent progress in aqueous zinc-ion batteries: a deep insight into zinc metal anodes. J. Mater. Chem. A.

[12] Yang Q, Li Q, Liu Z, Wang D, Guo Y (2020). Dendrites in Zn-based batteries. Adv. Mater..

[13] Yang Q, Liang G, Guo Y, Liu Z, Yan B (2019). Do zinc dendrites exist in neutral zinc batteries: a developed electrohealing strategy to in situ rescue in-service batteries. Adv. Mater..

[14] Jia H, Wang Z, Dirican M, Qiu S, Chan CY (2021). A liquid metal assisted dendrite-free anode for high-performance Zn-ion batteries. J. Mater. Chem. A.

[15] Song M, Tan H, Chao D, Fan HJ (2018). Recent advances in Zn-ion batteries. Adv. Funct. Mater..

[16] Li B, Zhang X, Wang T, He Z, Lu B (2022). Interfacial engineering strategy for high-performance Zn metal anodes. Nano-Micro Lett..

[17] Fu J, Cano ZP, Park MG, Yu A, Fowler M (2017). Electrically rechargeable zinc–air batteries: progress, challenges, and perspectives. Adv. Mater..

[18] Mainar AR, Iruin E, Colmenares LC, Kvasha A, de Meatza I (2018). An overview of progress in electrolytes for secondary zinc-air batteries and other storage systems based on zinc. J. Energy Storage.

[19] Chen A, Zhao C, Gao J, Guo Z, Lu X (2023). Multifunctional SEI-like structure coating stabilizing Zn anodes at a large current and capacity. Energy Environ. Sci..

[20] Lu X, Zhao C, Chen A, Guo Z, Liu N (2023). Reducing Zn-ion concentration gradient by SO_4_^2–^-immobilized interface coating for dendrite-free Zn anode. Chem. Eng. J..

[21] Xiao P, Li H, Fu J, Zeng C, Zhao Y (2022). An anticorrosive zinc metal anode with ultra-long cycle life over one year. Energy Environ. Sci..

[22] Xie X, Liang S, Gao J, Guo S, Guo J (2020). Manipulating the ion-transfer kinetics and interface stability for high-performance zinc metal anodes. Energy Environ. Sci..

[23] Gu J, Tao Y, Chen H, Cao Z, Zhang Y (2022). Stress-release functional liquid metal-MXene layers toward dendrite-free zinc metal anodes. Adv. Energy Mater..

[24] Zeng Y, Sun PX, Pei Z, Jin Q, Zhang X (2022). Nitrogen-doped carbon fibers embedded with zincophilic Cu nanoboxes for stable Zn-metal anodes. Adv. Mater..

[25] Cao L, Li D, Soto FA, Ponce V, Zhang B (2021). Highly reversible aqueous zinc batteries enabled by zincophilic–zincophobic interfacial layers and interrupted hydrogen-bond electrolytes. Angew. Chem. Int. Ed..

[26] Luo M, Wang C, Lu H, Lu Y, Xu BB (2021). Dendrite-free zinc anode enabled by zinc-chelating chemistry. Energy Storage Mater..

[27] Wang B, Zheng R, Yang W, Han X, Hou C (2022). Synergistic solvation and interface regulations of eco-friendly silk peptide additive enabling stable aqueous zinc-ion batteries. Adv. Funct. Mater..

[28] Zhang Q, Ma Y, Lu Y, Zhou X, Lin L (2021). Designing anion-type water-free Zn^2+^ solvation structure for robust Zn metal anode. Angew. Chem. Int. Ed..

[29] Lu J, Yang J, Zhang Z, Wang C, Xu J (2022). Silk fibroin coating enables dendrite-free zinc anode for long-life aqueous zinc-ion batteries. Chemsuschem.

[30] Xu J, Lv W, Yang W, Jin Y, Jin Q (2022). In situ construction of protective films on Zn metal anodes via natural protein additives enabling high-performance zinc ion batteries. ACS Nano.

[31] Liang S, Miao J, Shi H, Zeng M, An H (2022). Tuning interface mechanics via β-configuration dominant amyloid aggregates for lithium metal batteries. ACS Nano.

[32] Lu H, Zhang X, Luo M, Cao K, Lu Y (2021). Amino acid-induced interface charge engineering enables highly reversible Zn anode. Adv. Funct. Mater..

[33] Meng Q, Zhao R, Cao P, Bai Q, Tang J (2022). Stabilization of Zn anode *via* a multifunctional cysteine additive. Chem. Eng. J..

[34] Yang X, Li C, Sun Z, Yang S, Shi Z (2021). Interfacial manipulation via in situ grown ZnSe cultivator toward highly reversible Zn metal anodes. Adv. Mater..

[35] Kresse G, Furthmüller J (1996). Efficient iterative schemes for ab initio total-energy calculations using a plane-wave basis set. Phys. Rev. B.

[36] Kresse G, Furthmüller J (1996). Efficiency of ab-initio total energy calculations for metals and semiconductors using a plane-wave basis set. Comp. Mater. Sci..

[37] Blöchl PE (1994). Projector augmented-wave method. Phys. Rev. B.

[38] Perdew JP, Burke K, Ernzerhof M (1996). Generalized gradient approximation made simple. Phys. Rev. Lett..

[39] Grimme S, Antony J, Ehrlich S, Krieg H (2010). A consistent and accurate ab initio parametrization of density functional dispersion correction (DFT-D) for the 94 elements H-Pu. J. Chem. Phys..

[40] Momma K, Izumi F (2011). VESTA 3 for three-dimensional visualization of crystal, volumetric and morphology data. J. Appl. Crystallogr..

[41] Wang V, Xu N, Liu JC, Tang G, Geng WT (2021). VASPKIT: A user-friendly interface facilitating high-throughput computing and analysis using VASP code. Comput. Phys. Commun..

[42] Abraham MJ, Murtola T, Schulz R, Páll S, Smith JC (2015). GROMACS: high performance molecular simulations through multi-level parallelism from laptops to supercomputers. SoftwareX.

[43] Berendsen HJC, Grigera JR, Straatsma TP (1987). The missing term in effective pair potentials. J. Phys. Chem..

[44] Duan Y, Wu C, Chowdhury S, Lee MC, Xiong G (2003). A point-charge force field for molecular mechanics simulations of proteins based on condensed-phase quantum mechanical calculations. J. Comput. Chem..

[45] Wang J, Wolf RM, Caldwell JW, Kollman PA, Case DA (2004). Development and testing of a general amber force field. J. Comput. Chem..

[46] Menke A, Rex-Haffner M, Klengel T, Binder EB, Mehta D (2012). Peripheral blood gene expression: it all boils down to the RNA collection tubes. BMC Res. Notes.

[47] Lu T, Chen F (2012). Multiwfn: a multifunctional wavefunction analyzer. J. Comput. Chem..

[48] Martínez L, Andrade R, Birgin EG, Martínez JM (2009). PACKMOL: a package for building initial configurations for molecular dynamics simulations. J. Comput. Chem..

[49] Posch HA, Hoover WG, Vesely FJ (1986). Canonical dynamics of the Nosé oscillator: Stability, order, and chaos. Phys. Rev. A.

[50] Parrinello M, Rahman A (1981). Polymorphic transitions in single crystals: a new molecular dynamics method. J. Appl. Phys..

[51] Essmann U, Perera L, Berkowitz ML, Darden T, Lee H (1995). A smooth particle mesh Ewald method. J. Chem. Phys..

[52] York DM, Darden TA, Pedersen LG (1993). The effect of long-range electrostatic interactions in simulations of macromolecular crystals: a comparison of the Ewald and truncated list methods. J. Chem. Phys..

[53] Humphrey W, Dalke A, Schulten K (1996). VMD: visual molecular dynamics. J. Mol. Graph..

[54] Frisch MJ, Trucks GW, Schlegel HB, Scuseria GE, Robb MA (2016). Gaussian 16 Rev. C.01.

[55] Stephens PJ, Devlin FJ, Chabalowski CF, Frisch MJ (1994). Ab initio calculation of vibrational absorption and circular dichroism spectra using density functional force fields. J. Phys. Chem..

[56] Weigend F, Ahlrichs R (2005). Balanced basis sets of split valence, triple zeta valence and quadruple zeta valence quality for H to Rn: design and assessment of accuracy. Phys. Chem. Chem. Phys..

[57] Marenich AV, Cramer CJ, Truhlar DG (2009). Universal solvation model based on solute electron density and on a continuum model of the solvent defined by the bulk dielectric constant and atomic surface tensions. J. Phys. Chem. B.

[58] Gutowski M, Van Lenthe JH, Verbeek J, Van Duijneveldt FB, Chałasinski G (1986). The basis set superposition error in correlated electronic structure calculations. Chem. Phys. Lett..

[59] Li X, Yuan L, Liu D, Liao M, Chen J (2021). Elevated lithium ion regulation by a “natural silk” modified separator for high-performance lithium metal anode. Adv. Funct. Mater..

[60] Huang C, Zhao X, Hao Y, Yang Y, Qian Y (2022). Highly reversible zinc metal anodes enabled by protonated melamine. J. Mater. Chem. A.

[61] Huang C, Zhao X, Liu S, Hao Y, Tang Q (2021). Stabilizing zinc anodes by regulating the electrical double layer with saccharin anions. Adv. Mater..

[62] Lv Y, Zhao M, Du Y, Kang Y, Xiao Y (2022). Engineering a self-adaptive electric double layer on both electrodes for high-performance zinc metal batteries. Energy Environ. Sci..

[63] Qin R, Wang Y, Zhang M, Wang Y, Ding S (2021). Tuning Zn^2+^ coordination environment to suppress dendrite formation for high-performance Zn-ion batteries. Nano Energy.

[64] Xu W, Zhao K, Huo W, Wang Y, Yao G (2019). Diethyl ether as self-healing electrolyte additive enabled long-life rechargeable aqueous zinc ion batteries. Nano Energy.

[65] Deng C, Xie X, Han J, Tang Y, Gao J (2020). A sieve-functional and uniform-porous kaolin layer toward stable zinc metal anode. Adv. Funct. Mater..

[66] Zhang H, Guo R, Li S, Liu C, Li H (2022). Graphene quantum dots enable dendrite-free zinc ion battery. Nano Energy.

[67] He Q, Fang G, Chang Z, Zhang Y, Zhou S (2022). Building ultra-stable and low-polarization composite Zn anode interface *via* hydrated polyzwitterionic electrolyte construction. Nano-Micro Lett..

[68] Wang D, Li Q, Zhao Y, Hong H, Li H (2022). Insight on organic molecules in aqueous Zn-ion batteries with an emphasis on the Zn anode regulation. Adv. Energy Mater..

[69] Chen W, Guo S, Qin L, Li L, Cao X (2022). Hydrogen bond-functionalized massive solvation modules stabilizing bilateral interfaces. Adv. Funct. Mater..

[70] Yang H, Qiao Y, Chang Z, Deng H, Zhu X (2021). Reducing water activity by zeolite molecular sieve membrane for long-life rechargeable zinc battery. Adv. Mater..

[71] Hao J, Li X, Zeng X, Li D, Mao J (2020). Deeply understanding the Zn anode behaviour and corresponding improvement strategies in different aqueous Zn-based batteries. Energy Environ. Sci..

[72] Pan H, Shao Y, Yan P, Cheng Y, Han KS (2016). Reversible aqueous zinc/manganese oxide energy storage from conversion reactions. Nat. Energy.

[73] Sun W, Wang F, Hou S, Yang C, Fan X (2017). Zn/MnO_2_ battery chemistry with H^+^ and Zn^2+^ coinsertion. J. Am. Chem. Soc..

