# Supplementary material for: Synergistic “Anchor-Capture” Enabled by Amino and Carboxyl for Constructing Robust Interface of Zn Anode
Source: Nanomicro Lett. 2023 Aug 28;15:205. doi: 10.1007/s40820-023-01171-w (PMC10462588; doi:10.1007/s40820-023-01171-w)
Supplement: Supplementary file 1 — Supplementary file1 (PDF 1454 KB) [file 40820_2023_1171_MOESM1_ESM.pdf]

Supporting Information for

Synergistic "Anchor-Capture" Enabled by Amino and Carboxyl for Constructing Robust Interface of Zn Anode

Zhen Luo[1,2,] †, Yufan Xia[1,2,] †, Shuang Chen[1,2], Xingxing Wu[2], Ran Zeng[3], Xuan Zhang[2], Hongge Pan[1,4], Mi Yan[1,5], Tingting Shi[6,] *, Kai Tao[3,7], Ben Bin Xu[8,] *, Yinzhu Jiang[1,2,] *

[1]School of Materials Science and Engineering, Zhejiang University, Hangzhou 310027, People's Republic of China

[2]ZJU-Hangzhou Global Scientific and Technological Innovation Center, Zhejiang University, Hangzhou 311215, People's Republic of China

[3]State Key Laboratory of Fluid Power and Mechatronic Systems, Key Laboratory of Advanced Manufacturing Technology of Zhejiang Province, School of Mechanical Engineering, Zhejiang University, Hangzhou 310027, People's Republic of China

[4]Institute of Science and Technology for New Energy, Xi'an Technological University, Xi'an 710021, People's Republic of China

[5]State Key Laboratory of Baiyunobo Rare Earth Resource Researches and Comprehensive Utilization, Baotou Research Institute of Rare Earths, Baotou 014030, People's Republic of China

[6]Guangdong Provincial Engineering Technology Research Center of Vacuum Coating Technologies and New Energy Materials, Department of Physics, Jinan University, Guangdong, Guangzhou 510632, People's Republic of China

[7]Zhejiang-Israel Joint Laboratory of Self-Assembling Functional Materials; Hangzhou 311200, People's Republic of China

[8]Mechanical and Construction Engineering, Faculty of Engineering and Environment, Northumbria University, Newcastle upon Tyne, NE1 8ST, United Kingdom

†Z. Luo and Y. Xia contributed equally to this work.

*Corresponding authors. E-mails: yzjiang@zju.edu.cn (Yinzhu Jiang); ben.xu@northumbria.ac.uk (Ben Bin Xu); ttshi@email.jnu.edu.cn (Tingting Shi)

Supplementary Figures and Tables

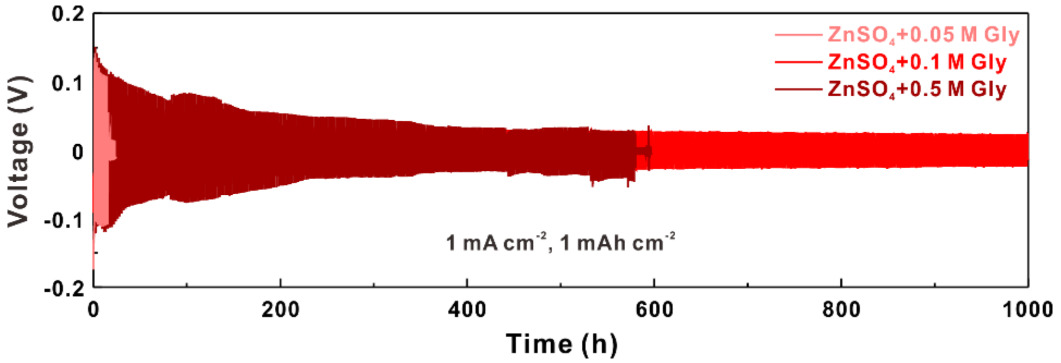

Fig. S1 The voltage profiles of Zn-Zn symmetric cells with 0.05/0.1/0.5 M Gly tested at the current density of 1 mA cm^{-2} with the areal capacity of 1 mAh cm^{-2}

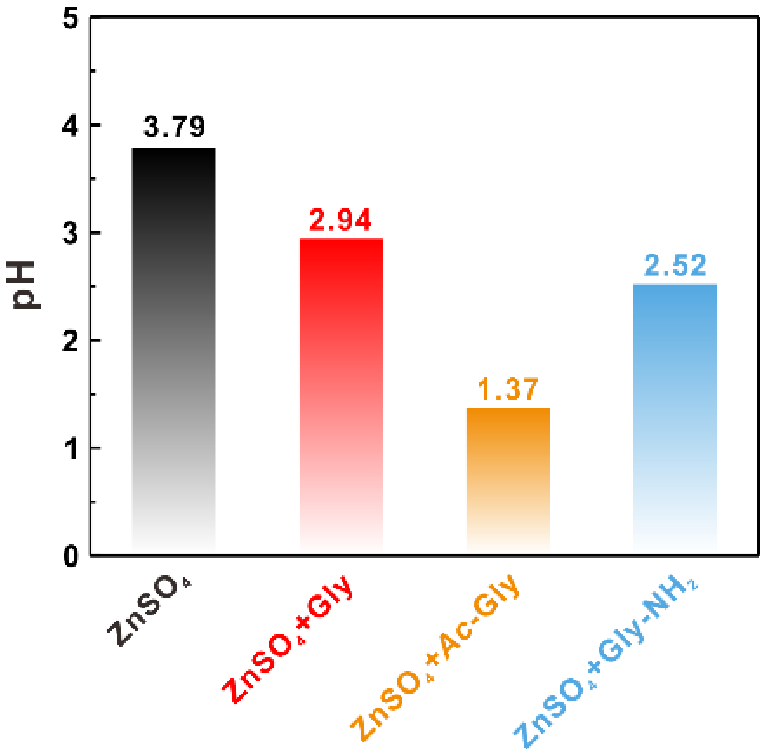

Fig. S2 The pH of bare $ZnSO_4$ electrolytes with/without Gly/*Ac*-Gly/Gly-NH_2

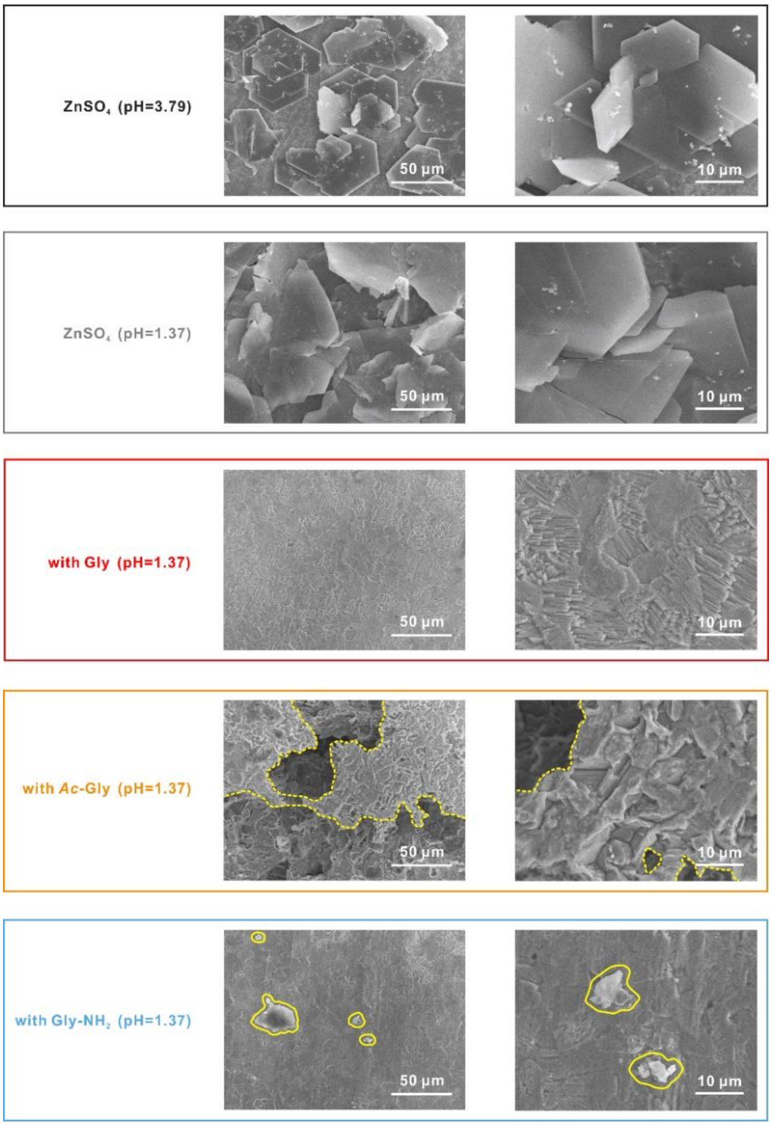

Fig. S3 SEM images of Zn plates surface morphologies soaked in different solutions for 7 days

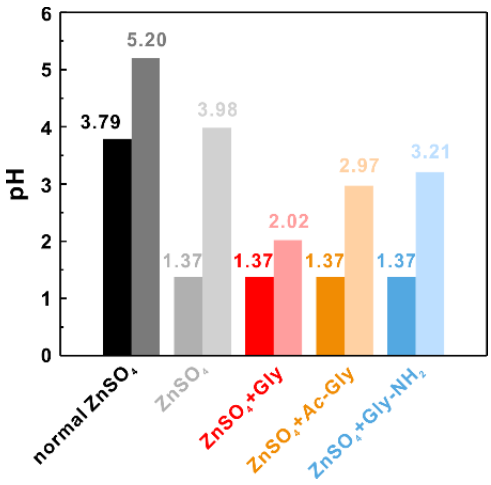

Fig. S4 The evolution of pH value before and after Zn plate immersed in different solutions for 7 days

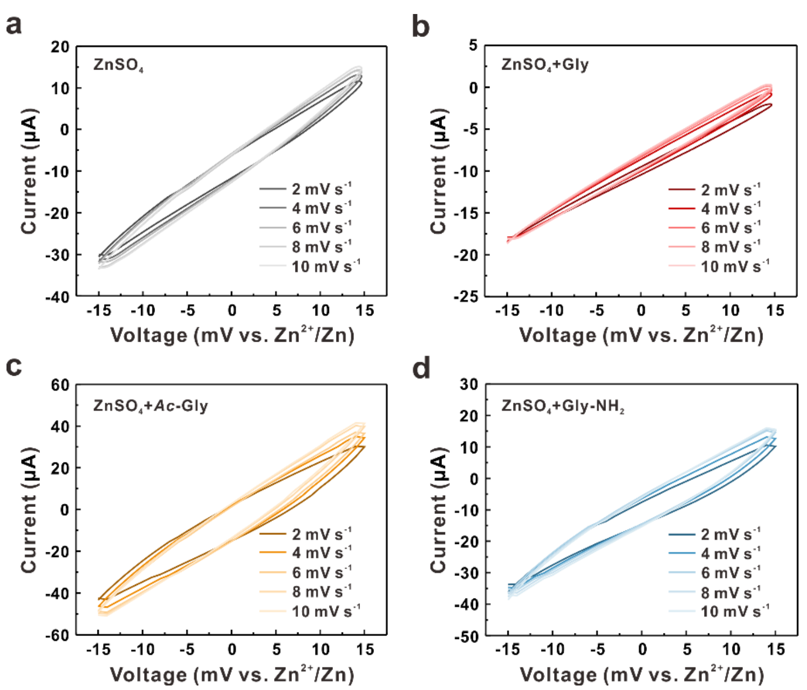

Fig. S5 CV curves of Zn-Zn symmetric cells at various scan rates from 2 to 10 mV s^{-1} in **a** bare $ZnSO_4$; **b** $ZnSO_4$+Gly; **c** $ZnSO_4$+*Ac*-Gly; and **d** $ZnSO_4$+Gly-NH_2 electrolytes

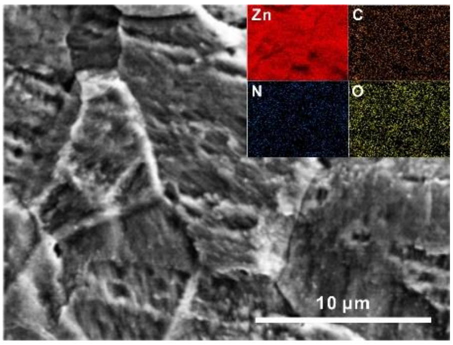

Fig. S6 SEM image of Zn plate soaked in Gly/H_2O solution for 7 days and the corresponding EDS mapping

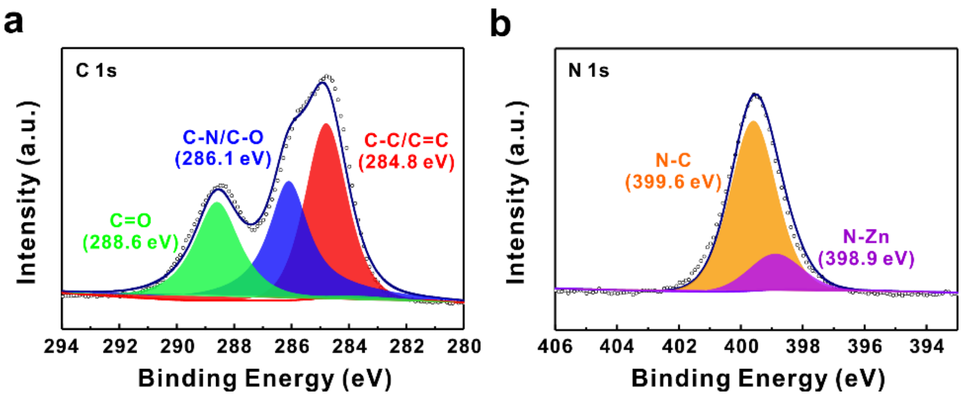

Fig. S7 The high-resolution XPS spectra of Zn plate soaked in Gly/H_2O solution for 7 days: **a** C 1s; **b** N 1s

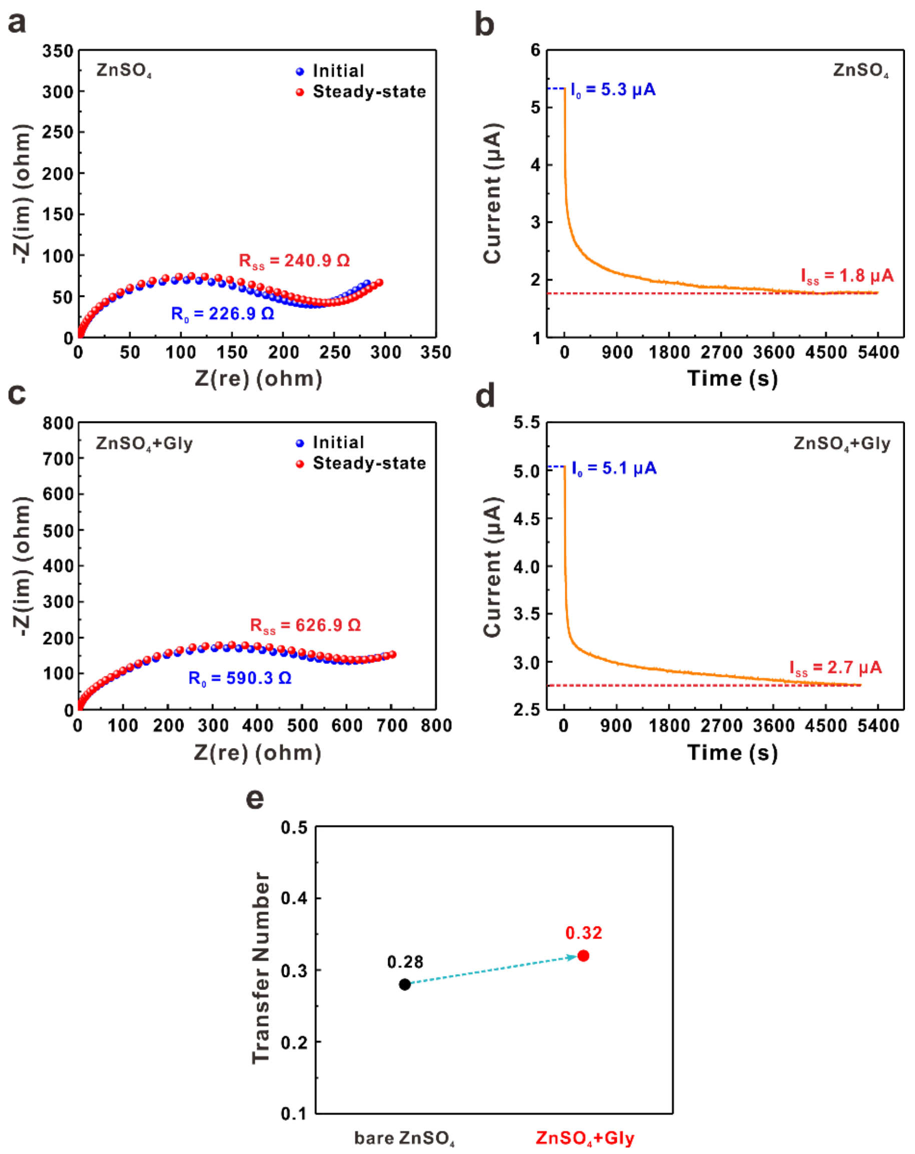

Fig. S8 EIS spectra of the Zn-Zn symmetric cells **a** without and **c** with Gly additive before and after polarization. The corresponding chronoamperograms of the Zn-Zn symmetric cells **b** without and **d** with Gly additive under the applied overpotential of 5 mV. **e** Calculated Zn^{2+} transfer numbers ($t(Zn^{2+})$) of $ZnSO_4$ electrolyte with/without Gly

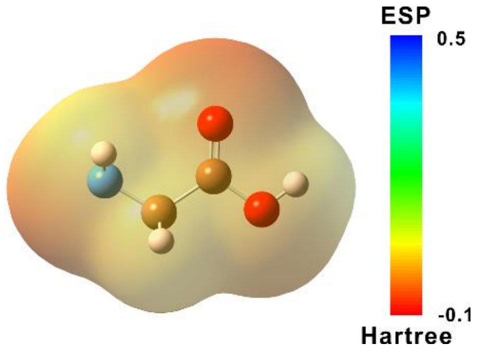

Fig. S9 Electrostatic potential mapping of Gly molecule

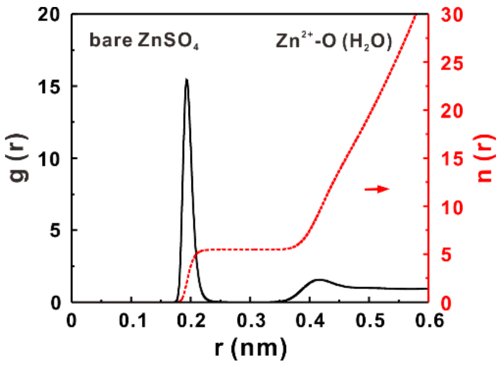

Fig. S10 Simulated RDF for Zn^{2+}-O (H_2O) in bare $ZnSO_4$ electrolyte

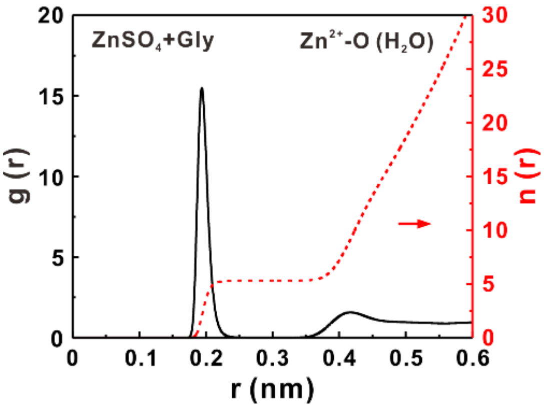

Fig. S11 Simulated RDF for Zn^{2+}-O (H_2O) in $ZnSO_4$+Gly electrolyte

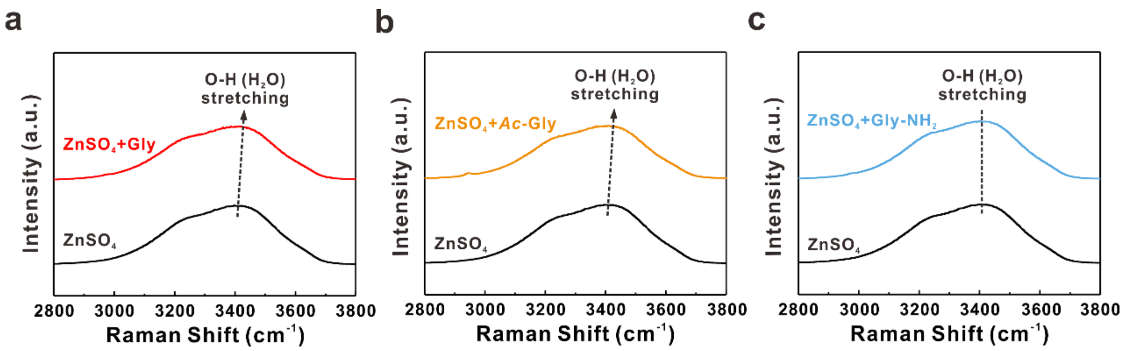

Fig. S12 Raman spectra for different electrolytes

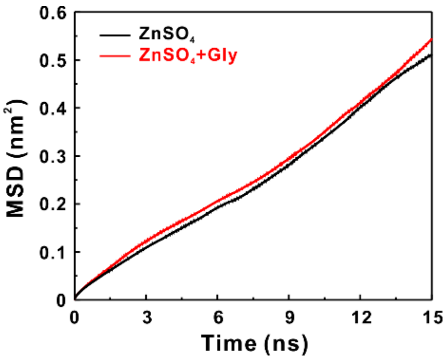

Fig. S13 MSD as a function of time under $ZnSO_4$ and Gly-containing electrolytes

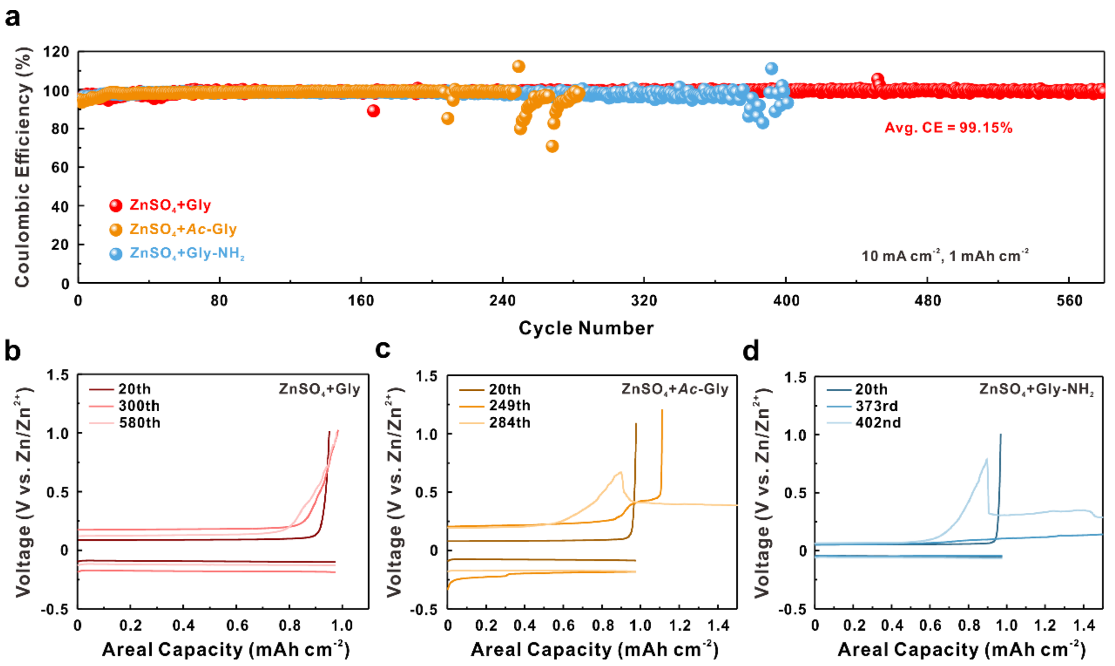

Fig. S14 **a** CE comparison of Zn-Ti cells in $ZnSO_4$+Gly/*Ac*-Gly/Gly-NH_2 electrolytes at 10 mA cm^{-2} and 1 mAh cm^{-2} and **b**, **c**, **d** corresponding voltage profiles at different cycles, respectively

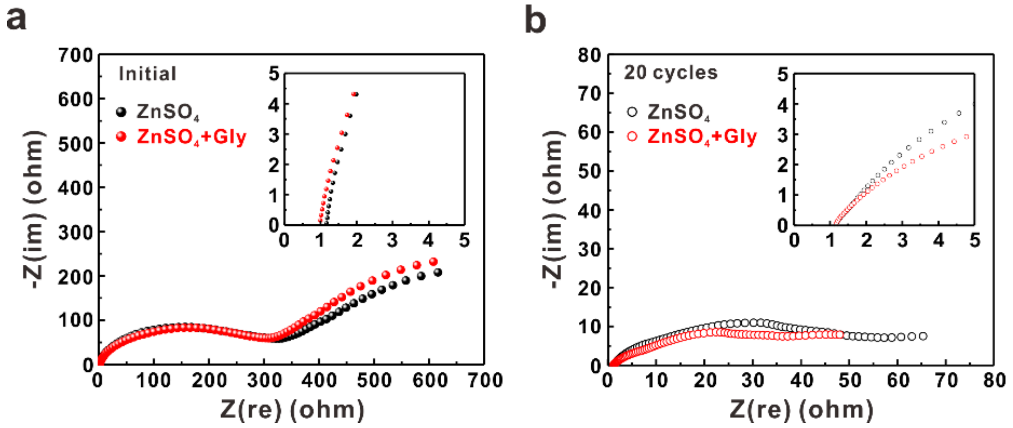

Fig. S15 EIS results of Zn-Zn symmetric cells using bare $ZnSO_4$ and $ZnSO_4$+Gly electrolytes **a** before and **b** after 20 cycles

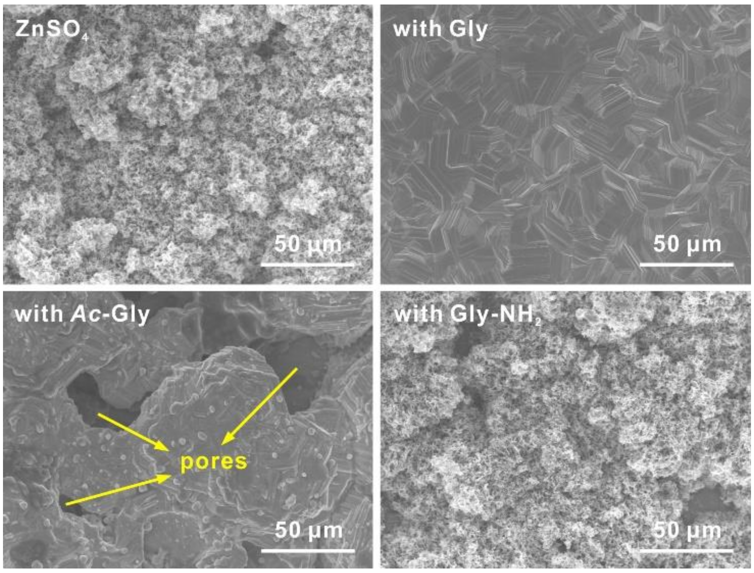

Fig. S16 The SEM images of Zn electrodeposition on Zn plates in different electrolytes for 2 h at 5 mA cm^{-2}

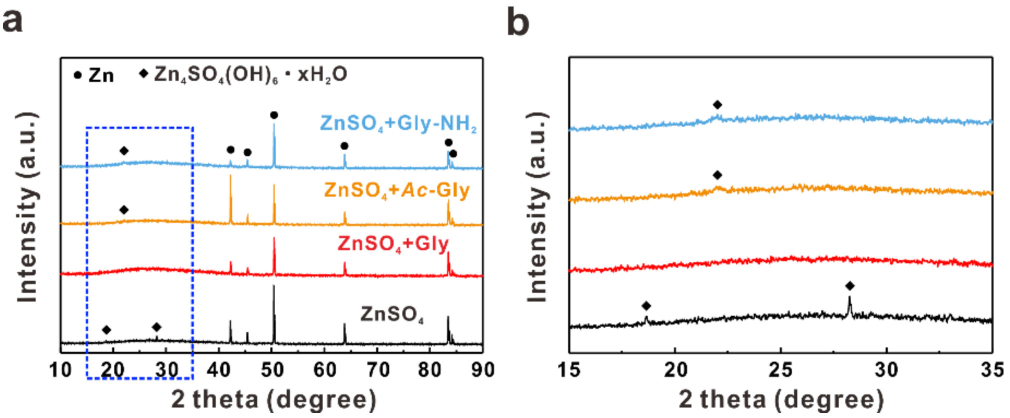

Fig. S17 a XRD patterns of Zn anode after 50 cycles in different electrolytes. **b** Partial XRD patterns at 2 theta among 15°-35°

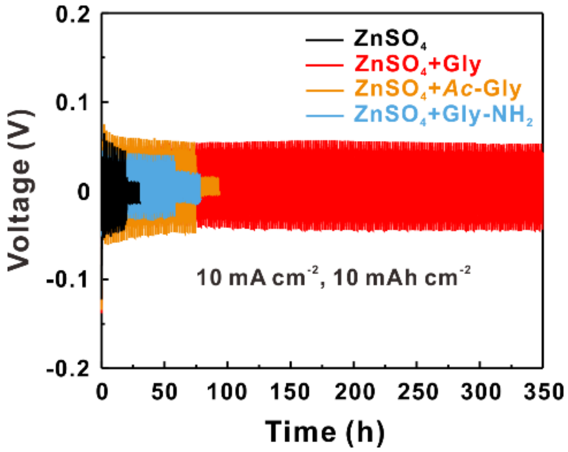

Fig. S18 The voltage profiles of Zn-Zn symmetric cells with/without Gly/*Ac*-Gly/Gly-NH_2 tested at the current density of 10 mA cm^{-2} with the areal capacity of 10 mAh cm^{-2}

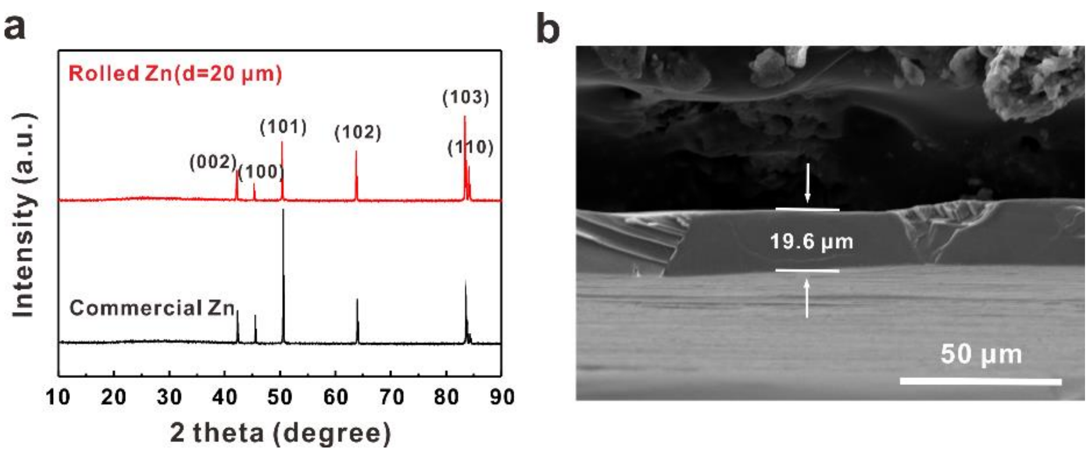

Fig. S19 **a** XRD patterns of commercial Zn plate and rolled Zn foil. **b** The cross-section SEM image of rolled Zn foil

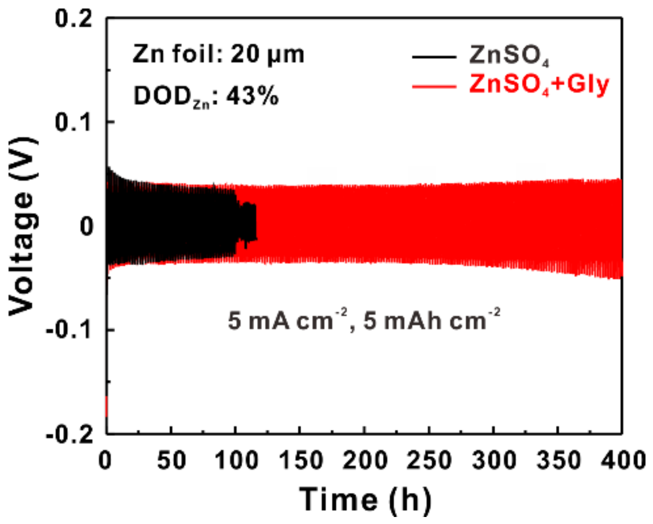

Fig. S20 The voltage profiles of Zn-Zn symmetric cells with/without Gly tested at the current density of 5 mA cm^{-2} with the areal capacity of 5 mAh cm^{-2} (rolled Zn foil as anode with the DOD_{Zn} of 43%)

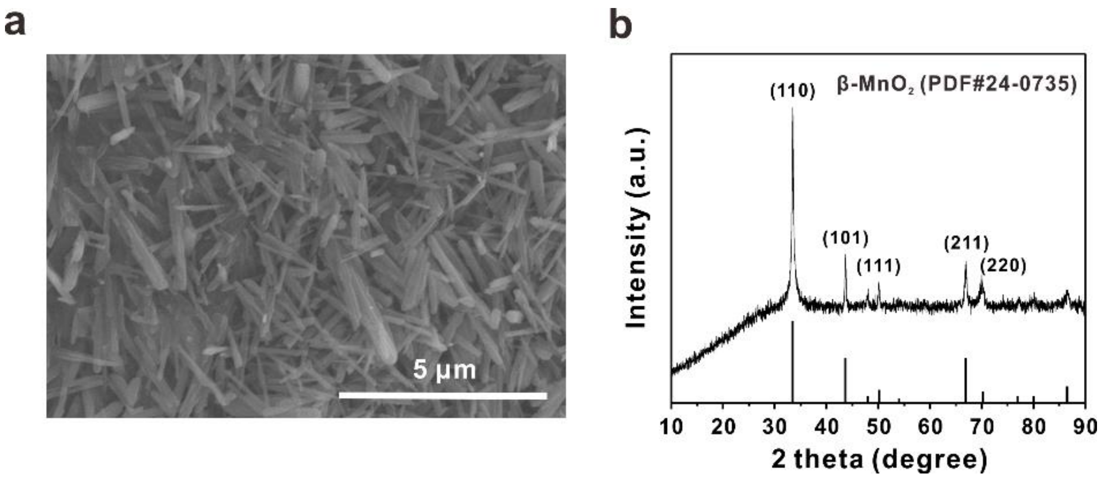

Fig. S21 **a** SEM image and **b** XRD pattern of β-MnO_2 cathode (PDF#24-0735)

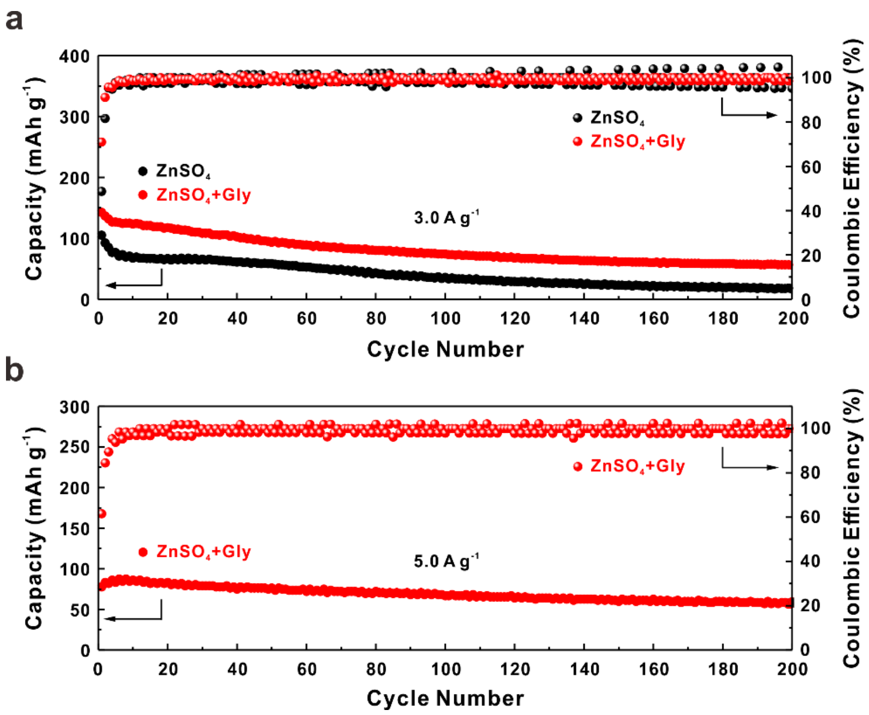

Fig. S22 Cycling performances of Zn-MnO_2 full cells at the current density of **a** 3.0 A g^{-1} and **b** 5.0 A g^{-1} with/without Gly. 0.1 M $MnSO_4$ was added into each electrolyte

Table S1 The corrosion current density (j_{cor}) and corrosion potential (V_{cor}) of Zn anodes cycled in different electrolytes derived from Fig. 1c

Electrolytes	Corrosion current density j_{cor} (mA cm^{-2})	Corrosion potential V_{cor} (V)
bare $ZnSO_4$	4.23	-0.975
$ZnSO_4$ + Gly	1.11	-0.974
$ZnSO_4$ + *Ac*-Gly	1.45	-0.975
$ZnSO_4$ + Gly-NH_2	1.30	-0.974

Table S2 The growth rate of current density between 50-200 s in different electrolytes derived from Fig. 2b

Electrolytes	Current density increased from 50-200 s (mA cm^{-2})	Growth rate (mA cm^{-2} s^{-1})
bare $ZnSO_4$	5.28	0.035
$ZnSO_4$ + Gly	2.75	0.018
$ZnSO_4$ + *Ac*-Gly	3.24	0.022
$ZnSO_4$ + Gly-NH_2	3.42	0.023
